# Population structure and gene flux of Listeria monocytogenes ST121 reveal prophages as a candidate driver of adaptation and persistence in food production environments

**DOI:** 10.1099/mgen.0.001397

**Published:** 2025-04-17

**Authors:** Ana Victoria Gutiérrez, Michaela Matthews, Maria Diaz, Thanh Le Viet, Leonardo de Oliveira Martins, Frieda Jørgensen, Heather Aird, Anaïs Painset, Nicolle Som, Oleksii Omelchenko, Evelien M. Adriaenssens, Robert A. Kingsley, Matthew W. Gilmour

**Affiliations:** 1 Quadram Institute Bioscience, Norwich Research Park, Norwich, UK; 2 UK Health Security Agency, Food, Water and Environmental Microbiology Laboratories and the Gastrointestinal Bacteria Reference Unit, London, UK; 3 University of East Anglia, Norwich, UK

**Keywords:** foodborne pathogen, hierarchical clustering, *Listeria monocytogenes* ST121, pangenome diversity, population structure, prophage

## Abstract

*Listeria monocytogenes* is a bacterial pathogen found in an increasing number of food categories, potentially reflecting an expanding niche and food safety risk profile. In the UK, *Listeria monocytogenes* sequence type (ST) 121 is more frequently isolated from foods and food environments than from cases of clinical listeriosis, consistent with a relatively low pathogenicity. In this study, we determined the evolution associated with the environmental persistence of a *Listeria monocytogenes* clone by investigating clone-specific genome features in the context of the ST121 population structure from international sources. To enable unambiguous comparative genomic analysis of ST121 strains, we constructed 16 new high-quality genome assemblies from *Listeria monocytogenes* isolated from foods, food environments and human clinical sources in the UK from 1987 to 2019. Our dataset was supplemented with additional UK and international genomes from databases held by the Institut Pasteur and the UK Health Security Agency. Time-scaled phylogenetic reconstruction revealed that clade-specific microevolution correlated with key characteristics that may confer adaptations important for success in the environmental niche. For example, a prophage designated LP-13-6 unique to a clade is associated with multi-year persistence in a food production setting. This prophage, observed in a strain that persisted for over a decade, may encode mechanisms facilitating environmental persistence, including the exclusion of other bacteriophages. Pangenome analysis provided insights into other candidate genetic elements associated with persistence and biocide tolerance. The comparative genomic dataset compiled in this study includes an international collection of 482 genome sequences that serve as a valuable resource for future studies to explore conserved genes, regulatory regions, mutations and variations associated with particular traits, such as environmental persistence, pathogenicity or biocide tolerance.

**Impact Statement**

This study advances the understanding of the phylogenetic structure and genomic dynamics of *Listeria monocytogenes* ST121, a prevalent sequence type (ST) associated with food and food-related environments. Key findings include the identification and description of genetic signature regions linked to sanitizer resistance, as well as clade-specific elements such as prophages, transposon and a genomic island, all of which are candidate mechanisms contributing to environmental adaptation, persistence and spread of ST121. Identifying clade-specific signature regions supports risk assessment by enabling prediction of a strain’s likelihood to persist in specific settings and providing opportunities for the development of more effective surveillance and control strategies. For example, monitoring high-risk food products, conducting focused inspections and microbial testing in environments with a history of *Listeria monocytogenes* ST121 presence can help prevent outbreaks. Additionally, using genomic tools to track ST121 strains that carry genetic markers linked to sanitizer resistance or other adaptive traits aids in controlling the spread of this pathogen. Ultimately, understanding how signature regions function and contribute to the resilience and spread of *Listeria monocytogenes* ST121 can inform public health interventions and improve food safety measures by enabling more effective responses to contamination and outbreaks.

## Data Summary

Data are available in the National Center for Biotechnology Information (NCBI) under BioProjects PRJNA837734 (Quadram Institute Bioscience), PRJNA248549 (UK Health Security Agency) and PRJEB12738 (Institut Pasteur).

The authors confirm that all supporting data and protocols have been provided within the article or through supplementary data files.

## Introduction

*Listeria monocytogenes* is widespread in the environment [1,2] and poses a substantial health risk to susceptible people when it contaminates food. It is particularly dangerous for individuals with compromised immune systems, individuals at the extremes of age and expectant mothers, where it can lead to adverse pregnancy outcomes including spontaneous abortion or stillbirth [3]. In 2022, despite the overall low rate of listeriosis cases across EU member states (0.62 per 100,000 population), the incidence reached the highest rate and number of cases since 2007, with an overall EU case fatality rate of 18.1% [4] and an even higher fatality rate for England and Wales of 23.1% [5].

The population structure of *Listeria monocytogenes* consists of four phylogenetic lineages (I, II, III and IV), each containing further diversification into clonal complexes (CCs) comprising one or multiple sequence types (STs). Strains of CCs within lineage II are frequently isolated from food and are commonly associated with contamination of food production environments (FPEs) [6]. *Listeria monocytogenes* strains, particularly those of ST121, have been found in various ready-to-eat food products such as dairy, turkey breast, fish and sandwiches [7,16] and are notable for their persistence in FPEs [10,13, 15, 17,25], where persisting clones may originate from external sources and migrate to additional settings [26]. Their survival is supported by favourable environmental conditions and adaptive traits, such as biofilm production, tolerance to high humidity and low-temperature settings, withstanding pH changes and high osmolarity and resistance to disinfectant biocides [27,31]. These adaptations support their persistence, complicating risk management efforts, and once present in FPEs, they can pose a significant challenge to ensuring food safety.

The *Listeria monocytogenes* genome is characterized by a generally stable core structure, with ~78% of coding sequences comprising conserved core genes [32]. Despite this stability, the genome also includes mobile chromosomal elements, such as prophages, transposons and genomic islands [29], which can be located in hotspots characterized by a high frequency of genetic variation and recombination [32,33]. Prophages, temperate bacteriophages inserted into the bacterial genome, account for a significant portion of the total accessory gene pool [10]. They play a role in bacterial evolution by exerting selective pressure and facilitating horizontal gene transfer [34], but also influencing the pathogenic and physiological traits of bacterial populations [35,36]. In *Listeria monocytogenes*, prophage acquisition often enhances bacterial fitness rather than incorporating virulence genes [10,34, 37] and may confer resistance to subsequent bacteriophage infections [38,39].

We investigated the population structure of *Listeria monocytogenes* ST121 in order to identify temporally and geographically localized phylogenetic strains. Clade and strain-specific microevolutionary events were identified, and their potential for conferring mechanisms that drive persistence was assessed. We explored the diversification of the ST121 clade and the temporal signatures of gene flux. In the context of these new insights into the population structure, we tested the hypothesis that microevolutionary signatures of environmental adaptation were temporally associated with the introduction of a strain into a factory site, where it has persisted for the past 8 years [16,40].

## Methods

### Biocide susceptibility testing

The minimum inhibitory concentration (MIC) of benzalkonium chloride (BC) was assayed for 17 *Listeria monocytogenes* strains that are part of this study (07-032, BL87-028, BL89-019, BL89-020, 399454, 246242, 163381, 241745, 457441, 389690, 535305, 396044, 246255, 259390, 754322, 757923 and LS12-859) following an adapted version of the agar dilution antimicrobial susceptibility method [41]. Briefly, lysed horse blood-Mueller–Hinton agar (LHB-MHA) was supplemented with BC with alkyl distribution from C8H17 to C18H37 (Merck) to achieve two-fold dilutions of BC with final concentrations ranging from 2.5 to 20 µg ml^−1^. Using a 96-well pin replicator [Molecular Devices (UK) Ltd], agar plates were inoculated with 10^4^ c.f.u. per strain. Control LHB-MHA plates were inoculated before and after the BC-containing plates to ensure there was no contamination or significant antimicrobial carryover during the inoculation. Plates were incubated at 37 °C for 20 h, and MIC was recorded.

### DNA extraction and sequencing

DNA was extracted from 17 bacterial strains (this study), following 2 different methods: DNA used for short-read sequencing was extracted from 800 µl of overnight culture using the automated Maxwell® RSC 48 instrument and the Maxwell® RSC Cultured Cells DNA (Promega) kit following the manufacturer’s instructions; DNA used for long-read sequencing was purified from 1 ml of overnight culture using the Fire Monkey High Molecular Weight DNA extraction kit (RevoluGen Ltd) after treating the cells with 20 mg.ml^−1^ lysozyme (Rocher®) and 1.2% Triton X-100 (Merck) for 30 min at 37 °C and 180 r.p.m. shaking. DNA was quantified using the QuantiFluor® dsDNA System and GloMax® Discover Microplate Reader (Promega). Paired-end short-read sequencing libraries were prepared with the Illumina DNA Prep kit, and 150 bp paired-end reads were sequenced using the Illumina NextSeq 500 instrument with a NextSeq 500/550 Mid Output Kit v2 (300 cycles) (Illumina, Inc.). Long-read sequencing libraries were prepared using the nanopore ligation sequencing kit (SQK-LSK109) in combination with the native barcodes (EXP-NBD196) and sequenced using a SpotON flow cell (R9.4.1) and a MinION sequencing device. Data acquisition was performed using MinKNOW v3.1.8 software [42]. Base calling and adapter trimming were done using the high-accuracy mode of the basecaller Guppy 5.0.12+eb1 a981 (Oxford Nanopore Technologies).

### Genome assemblies and analysis

Hybrid assemblies of strains 07-032, BL87-028, BL89-019, BL89-020, 399454, 246242, 163381, 241745, 457441, 389690, 535305, 396044, 246255, 259390, 754322 and 757923 were produced using long and short reads. Long reads were adapter trimmed with Porechop v0.2.3 [43] and filtered with filtlong v0.2.1 [44] to keep the 95% of the best reads longer than 6,000 bp. Contamination of the filtered reads was assessed using kraken2 v2.1.1 [45] against the kraken2 database Standard [46]. A long-read assembly was produced using the filtlong filtered reads as input to the Flye assembler v2.7 [47] with the nano-raw mode. Long reads were filtered again with filtlong to keep reads longer than 1,000 bp, and the resulting reads were used to polish the circular long-read assemblies using medaka v1.4.3 [48]. Paired-end short reads were filtered with trimmomatic v0.38.0 [49] using default parameters with a sliding window of 4 bp and quality of 20 and inspected for contamination using kraken2 v2.1.1. The long-read assembly was further polished with the filtered short reads using pilon v1.24 [50] with the following nextflow script [51]. Complete, circularized genomes were submitted to the BIGSdb-Lm database for cgMLST type assessment [52] and are publicly available at https://doi.org/10.5281/zenodo.14906828.

Using default parameters, Fastq files from the UKHSA dataset underwent trimming using TrimGalore v0.4.3 [53]. Next, the quality of the resulting paired-end sequences was assessed using FastQC v0.11.8 [54], sequences were filtered based on a GC content above 39 mol% and N bases in a proportion higher than 5%. Assemblies were constructed using shovill v1.1.0 [55] with SPAdes v3.14 as assembler [56].

Assemblies from private and public collections of the Institute Pasteur’s BIGSdb [41] were downloaded on 22 September 2022.

Ragout [57] was used on the 482 assemblies to order contigs using 7 *Listeria monocytogenes* reference genomes (NZ_CP035187.1, NZ_CP028183.1, NC_021837.1, NZ_CP041211.1, NZ_CP016213.1, NC_021823.1 and NZ_CP023752.1).

### Phylogenetic reconstruction and population structure

A total of 482 *Listeria monocytogenes* ST121 genomes were included in the phylogenetic analysis [59 human isolates, 417 isolates from food and environment (FPEs) and 6 isolates from unknown sources; Dataset S1, available in the online Supplementary Material]. This dataset included 17 genomes from a collection at the Quadram Institute Bioscience (this study), collected between 1987 and 2019, 16 of which were complete genomes. These genomes were placed in phylogenetic context alongside 309 publicly available genomes gathered through UKHSA surveillance between 2008 and 2022 (accessed 18 June 2022) and 155 genomes from private and public collections spanning 1996–2020 (accessed 22 September 2022) via the Institute Pasteur’s BIGSdb [41] (http://bigsdb.pasteur.fr/listeria).

Assemblies of the 482 genomes were aligned to the reference genome CFSAN054109 (GCA_003030165.1) using Snippy v3.2 [58]. The reference genome was masked for repeat regions using BEDTools v2.29.0 [59], and a minimum base quality of 20, read coverage of 10× and 90% read concordance were used as criteria for variant calling. The alignment allowed the identification of SNPs within the core genome, as compared to the reference genome. Both monomorphic and variant sites were used to determine the total core genome size, which was computed using snp-sites v2.5.1 [60]. Gubbins v2.4.1 [44] was employed to detect potential recombination regions, and any identified regions were masked from the sequences. The recombination-purged variant sites were obtained by only including sites with A, C, G or T using snp-sites v2.5.1 [60]. RaxML v8.2.4 [61] was used to generate maximum likelihood phylogenetic trees using the sequence alignment for these sites, employing the GTRCAT model. Finally, IQ-TREE v2.2.6 [62] was used to correct for ascertainment bias using the -fconst flag with both the full alignment and N sites as input, employing the HKY+G model.

To determine the root of the phylogeny, the same phylogenetic reconstruction approach was applied separately including the genome 07-029 (BioSample: SAMN28229471) as the outgroup.

The population structure and nomenclature of clades were defined using Fastbaps [63], with Bayesian Analysis of Population Structure (BAPS) as the prior hyperparameter. This approach generated two levels of genetic variation based on the recombination-purged alignment that was utilized to construct the IQ-TREE phylogeny. After the first partition level was generated, a second level, conditioned on subset phylogenies (clades 1 to 13), resulted in hierarchical partitioning that maximized the BAPS likelihood.

### Bayesian dating

A time-scaled phylogeny was constructed in R using the BactDating v1.0.6 package [64], with the recombination-purged IQ-TREE tree, adjusted for bias correction, as input [65]. Branch lengths were rescaled from substitutions per site into units of substitutions, as required by BactDating. The number of sites used to build the tree included the sum of the alignment length and the count of the invariant (monomorphic) sites.

The molecular clock rate signal was assessed through linear regression of the root-to-tip distance and isolation dates, manually employing an appropriate root position as described above. For inference of ancestral node dates, the Bayesian Markov chain Monte Carlo algorithm in BactDating was run for 10^7^ iterations with the additive relaxed clock model to ensure convergence, resulting in an effective sample size above 100 for mu, sigma and alpha parameters according to the coda package [66].

### Pangenome analysis

Assemblies of the 482 genomes were annotated using Bakta v1.6.1 [67]. Next, we constructed the pangenome using Roary v3.13.0 [68], with the presence and absence of genes identified without splitting paralogous genes. Pangenome sequences are available at https://doi.org/10.5281/zenodo.14906828. Core genes were defined as those present in 99% of genomes (*n*=478) and accessory genes as those present in less than 99% of the genomes and within the accessory gene subcategories such as shell genes (ranging from 15% to <99% of the isolates) and cloud genes (less than 15% of the isolates) [69]. A rarefaction curve was constructed [69] to evaluate if the ST121 pangenome was open or closed, based on Heaps’ law statistical model proposed by Tettelin *et al*. [70], where the pangenome is considered open when *γ*>0 and closed when *γ*<0 [69].

For comparative analysis, we categorized the genomic composition according to their origin. First, we constructed three reference-based gene databases: chromosome, plasmid and phage. This was done using 108 completely assembled *Listeria monocytogenes* reference genomes comprising 56 STs, 41 *Listeria* plasmids and 30 *Listeria* phage sequences obtained from the NCBI (Dataset S2). We extracted plasmid sequences from the 108 reference assemblies and examined both the plasmid and chromosomal sequences for the presence of prophages using Vibrant v1.2.1 [71]. With the exception of pCFSAN008100, all plasmids were found to be free of prophage. The coordinates of identified prophages were then used to extract prophage sequences using custom R scripts.

The prophage-free chromosome gene database, referred to as ‘chromosome’, consisted of 108 sequences. The prophage-free plasmid gene database, referred to as ‘plasmid’, included 65 sequences (23 extracted from complete genomes and 42 downloaded from the NCBI). The phage gene database, referred to as ‘phage’, contained 295 sequences (264 extracted from reference chromosomes, 1 extracted from a plasmid and 30 downloaded from the NCBI). All sequences were annotated using Bakta v1.6.1 [67], and each reference gene database was individually processed with Roary v3.13.0 [68]. The resulting chromosome, plasmid and phage pangenome FASTA files (available at https://doi.org/10.5281/zenodo.14906828) were used as input to categorize the genes from the dataset of 482 genomes. The categorization was performed using nucmer, an extension of the MUMmer v3.2.3 package [72], based on a threshold of more than 90% nt sequence identity and length of the reference and query sequences [65]. Gene families displaying less than 90% sequence identity to genes in the customized reference gene databases were categorized as ‘undefined’.

Locus tags of classified core genes (chromosome, phage and plasmid) were manually mapped to a complete genome (757923). Core phage-related genes located contiguously were (i) aligned to the nt collection database on the NCBI using the web-based Basic Local Alignment Search Tool (blast) [73] and (ii) compared against the Infrastructure for a Phage Reference Database (INPHARED) (1 August 2024) using the tool mash v2.0 [74] with the get_closest_relatives.pl script [75].

### Presence/absence of *Listeria* genetic determinants

From the genome assemblies of the ST121 collection, we explored the presence of genes associated with various functional categories in the BIGSdb-Lm database, including virulence (92 loci), resistance (34 loci, including resistance to metal, disinfectant and antibiotics), stress islands (7 loci), *Listeria* genomic Islands (117 loci), motility (31 loci), rhamnose (8 loci) and the *sigB* operon (8 loci). This analysis utilized the BIGSdb-Lm v1.47.3 database (https://bigsdb.pasteur.fr/listeria/, accessed on 19 September 2024) and was conducted using the blastn algorithm, as previously described [52].

### Pangenome-wide association studies

To identify genes uniquely present within hierarchical clades, a pangenome-wide association study (pan-GWAS) was conducted using Scoary v1.6.16 [76]. Correlations were performed on groups with ≥9 isolates, using a gene presence–absence matrix and a trait matrix. Each genome’s traits was categorized into binary categories, e.g. belonging to a hierarchical clade or not, represented as ‘1’ or ‘0’. A significant association was determined based on genes that displayed a specificity of ≥90% and sensitivity of ≥90% with an adjusted *P*-value<0.05. Adjusted *P*-values are reported for Bonferroni’s and Benjamini–Hochberg’s method for multiple comparison correction [76]. We do not use Scoary to infer causal associations along the tree since we provide the population structure through the BAPS clusters. Clades 1.1, 1.2, 1.3, 2.1, 3.1, 4.1, 5.1, 6.1, 8.2, 9.1, 9.2, 10.1, 11.1, 11.2, 13.1, 13.2 and 13.4, each with less than nine isolates, were excluded from the analysis.

The same approach was followed to identify genes specific to BC-resistant strains.

### Signature region analysis

A complete (757923) and four draft genomes (79397_130, 1838_H094800054, 1537877 and 1594542), representative of each clade in which significant gene association (*P*-value<0.05) was found by pan-GWAS, were used for mapping to clades 13.6 (757923), 1.4 (79397_130), 8.1 (1838_H094800054), 11.4 (1537877) and 12.2 (1594542) to assess their contiguity and spatial distribution. Additionally, the 757923 genome was utilized for mapping phenotypic resistance to BC. Annotations of hybrid genomes and draft genomes used for mapping signature regions are publicly available at https://doi.org/10.5281/zenodo.14906828. Groups of genes with statistically significant associations to a clade or BC resistance and located in the same genomic region were defined as signature regions. Genomic maps were generated with the web tool Proksee [77] and SnapGene® Viewer v6.0.2, followed by figure integration using Inkscape v0.92.4. Relatedness of signature regions to known phages was determined against the INPHARED (1 August 2024) using the get_closest_relatives.pl script [75], followed by annotation correction with Pharokka v1.3.2 [78] and homology comparison with clinker v0.0.23 [79]. The putative *attL* site, indicative of viral and host junction, was identified with the web tool PHASTEST [80]. Signature regions were aligned to the NCBI nt collection database using the web-based blast [73], and hits with a query coverage below 70% were excluded.

### Sequence analysis

aa alignments were performed using MAFFT (Multiple Alignment using Fast Fourier Transform) v7.525 [81] through the EMBL-EBI interface [82,83]. Conserved aa residues were shaded using Boxshade v3.3 [84]. To identify sequence motifs, we utilized the GenomeNet Bioinformatics Tool – MOTIF [85], incorporating searches within the NCBI-CDD and Pfam databases.

### Hotspots

Eleven hotspots (*lmo0061-lmo0075*, *lmo0137-lmo0152*, *lmo0293-lmo0296*, *lmo0301-lmo0314*, *lmo0377-lmo0382*, *lmo0432-lmo0436*, *lmo0458-lmo0480*, *lmo1096-lmo1126*, *lmo2025-lmo2028*, *lmo0443-lmo0449* and *lmo1702-lmo1703*) and three prophage insertion sites (*lmo1750-lmo1751*, *lmo1263* and *lmo0271-lmo0272*) [14,29, 32] were investigated in 16 ST121 high-quality genomes. Homologous EGD-e anchor genes were identified using Nucmer [72] based on a nt sequence identity threshold of more than 90%. Variations of the genetic regions between isolates were compared and illustrated by links between homologous genes based on percentage identity, as determined by clinker v0.0.23 [79].

## Results

### Time-scaled phylogeny of 482 ST121 genomes reveals the earliest emerged clade dating back to 1912

The population structure of *Listeria monocytogenes* ST121 was studied with a panel of 482 ST121 genomes from strains isolated in 13 countries between 1987 and 2022. This included 16 contiguously assembled genome sequences (hybrid assemblies) from an unpublished collection at Quadram Institute Bioscience (Dataset S1). The genomes within the ST121 panel varied in length from 2.85 to 3.3 Mbp, comprising 2,768 to 3,200 predicted coding sequences, with G+C content ranging from 37.7 mol% and 38.1 mol% (Dataset S1).

**Fig. 1.**
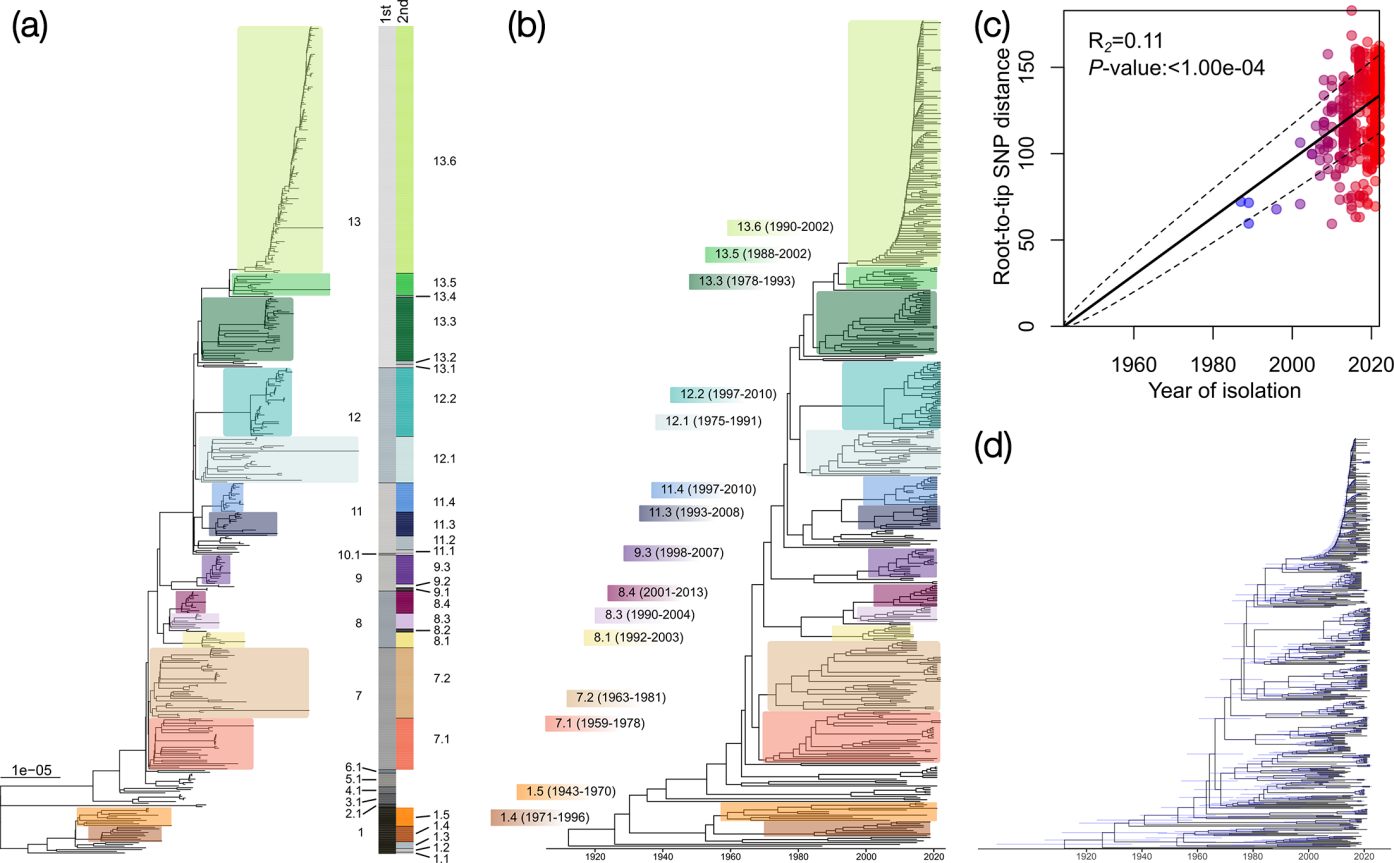
Population structure and time-calibrated phylogenetic tree of *Listeria monocytogenes* ST121. (a) A rooted maximum likelihood tree, corrected for ascertainment bias, was constructed based on a recombination-purged SNP core alignment of 482 *Listeria monocytogenes* isolates, using CFSAN054109 (GCA_003030165.1) as the reference genome. The clade nomenclature defining the population structure (1st, first-level and 2nd, second-level hierarchical clustering) highlights second-level clades, with those containing ≥9 isolates marked in distinct colours. The scale bar represents branch length measured in substitutions per site. (b) Time-scaled phylogeny constructed using BactDating. Labels and the dates of the common ancestor (±95% credibility intervals) are colour coded to match the clade clusters in the phylogeny. (c) Root-to-tip distance was calculated and displayed as a colour gradient, ranging from red (larger root-to-tip distance, more recent isolates) to blue (shorter root-to-tip distance, older isolates). The positive correlation between the number of accumulated SNPs and the year of collection, depicted by a continuous line, indicates a strong temporal signal (*P*-value 0.0001), with dashed lines representing 95% credibility intervals. (d) Inferred dated phylogeny, with shaded bars representing a 95% credibility interval for each node.

To determine the population structure of *Listeria monocytogenes* ST121, a two-level hierarchical Bayesian approach was used, based on the core SNP alignment and phylogeny of the 482 genomes (Fig. 1a). This clustering served as a guide for understanding the diverse clades and subclades within *Listeria monocytogenes* ST121, facilitating discussions within the context of this study. Notably, the clustering was derived from a phylogeny built on core genome SNPs purged of recombination, optimizing that groupings reflect true vertical evolutionary relationships rather than being significantly influenced by mobile genetic elements. Hierarchical clustering delineated 13 main clades in the first level, labelled as follows: clade 1 (27 isolates from Europe and South America), clade 2 (2 isolates from Mexico), clade 3 (6 isolates from Poland), clade 4 (4 isolates from Europe), clade 5 (8 isolates from the UK and the USA), clade 6 (2 isolates from Poland), clade 7 (71 isolates from Europe, the USA and Chile), clade 8 (33 isolates from Europe and Chile), clade 9 (21 isolates from the UK), clade 10 (1 isolate from the UK), clade 11 (41 isolates from Europe and Chile), clade 12 (67 isolates from Europe) and clade 13 (199 isolates from Europe and West Africa) (Fig. 1a, Dataset S1 and Fig. S1).

At the second level, 32 subclades were identified (1.1–1.5, 2.1, 3.1, 4.1, 5.1, 6.1, 7.1–7.2, 8.1–8.4, 9.1–9.3, 10.1, 11.1–11.4, 12.1–12.2 and 13.1–13.6), from which clades 1.1, 10.1 and 13.4 were represented by a single isolate (singletons) (Fig. 1a).

In addition, a time-scaled phylogeny was constructed using BactDating [64] to estimate when clades emerged (Fig. 1b). Root-to-tip regression analysis indicated a temporal signal in the accumulation of SNPs within *Listeria monocytogenes* ST121 (Fig. 1c, d). Amongst the 32 subclades, the earliest emerged clades were 1.2 [estimated to emerge in the year 1912 (95% confidence interval (CI) 1885–1934)], 1.3 [1936 (95% CI 1946–1980)], 1.5 [1959 (95% CI 1943–1970)] and 4.1 [1963 (95% CI 1949–1974)]. In contrast, clade 9.1 emerged more recently in 2019 (95% CI 2017–2020), followed by clade 2.1 in 2011 (95% CI 2003–2015) and clade 9.2 in 2010 (95% CI 2004–2013) (Table S1).

**Table 1.** Characteristics of signature regions associated with hierarchical clades and BC resistance in ST121

Signature region	No. of CDS*	Genome mapped	Type of element	GC content	Position in chromosome	Closest relative†						blastn‡		Insertion site
						Description	Mash distance	Identity (%)	Genus	Subfamily	Family	Identity (%)	Coverage (%)	
1.4(LP-01–4)	39	79397	Prophage	41.1	1749145–1802887	Unknown	–	–	–	–	–	–	–	Hotspot *lmo1702/lmo1703*
8.1_1	7	1838_H094800054	Prophage	36.5	685516–714006	*Listeria* phage FHC174-PLM34	0.0183741	98.16259	Unclassified	Unclassified	Unclassified	99.83	74	tRNA-Ser-CGA
8.1_2	5	1838_H094800054	Prophage	35.5	1288696–1330574	*Listeria* phage FHC15313-PLM26	0.0338037	96.61963	Slepowronvirus	Trabyvirinae	Unclassified	95.58	79	tRNA-Arg-TCT
						*Listeria* phage LP-101	0.0723061	92.76939	Slepowronvirus	Trabyvirinae	Unclassified	93.38	73	
11.4_1	24	SRR17120467	Prophage	34.9	1287356–1329450	*Listeria* phage LP-101	0.054149	94.5851	Slepowronvirus	Trabyvirinae	Unclassified	96.85	59	tRNA-Arg-TCT
						*Listeria* phage FHC15313-PLM26	0.0556462	94.43538	Slepowronvirus	Trabyvirinae	Unclassified	92.56	64	
11.4_2 (LGI-4)	8	SRR17120467	Genomic island	29.4	2225329–2232981	Unknown	–	–	–	–	–	–	–	tRNA-Leu-GAG
12.2(LP-12–2)	70	SRR18333768	Prophage	36.7	2776524–2822085	*Listeria* phage PSU-VKH-LP041	0.161822	83.8178	Unclassified	Unclassified	Unclassified	79.46	57	tRNA-Thr-GGT
						*Listeria* phage B054	0.164662	83.5338	Unclassified	Unclassified	Unclassified	79.16	67	
13.6(LP-13–6)	17	SRR9298670	Prophage	41.5	1809040–1859430	Unknown	–	–	–	–	–	–	–	*lmo1750/lmo1751*
BC^R^	5	SRR7167591	Transposon	33.61	1571155–1575679	Tn6188	–	–	–	–	–	99.96	100	*lmo1549*

a*Statistically associated (*P*-value below 0.05) by Scoary.

b†Prophage closests relative metrics were determined with INPHARED, which estimates identity percentages from Mash distance.

c‡Comparison between the signature region and closest relative using the nucleotident collection database on the NCBI.

BCR, BC resistant; CDS, coding sequence.

Clade 13.6 primarily included isolates from the UK collected during a study of a specific FPE (‘Company X’) and food produced therein such as sandwiches and salads [16], along with one isolate from Poland. This clade emerged around 1997 (95% CI 1990–2002), alongside clade 13.5, which emerged in 1996 (95 % CI 1988–2002) (Fig. 1d) and included isolates from Estonia, Poland and France and a subset causing clinical cases in the UK (Fig. S1).

Isolates from fish, seafood and meat were not clustered in a single clade but distributed in multiple clades. However, in comparison, clade 9.3 included isolates from an outbreak investigation involving cooked chicken in the UK [86], and clade 13.3 contained isolates from human breast milk, which has been associated with severe acute malnutrition in West Africa [87,88] (Dataset S1).

### Pangenome composition, core and accessory gene distribution and prophage-related insights for *Listeria monocytogenes* ST121 dynamics

**Fig. 2.**
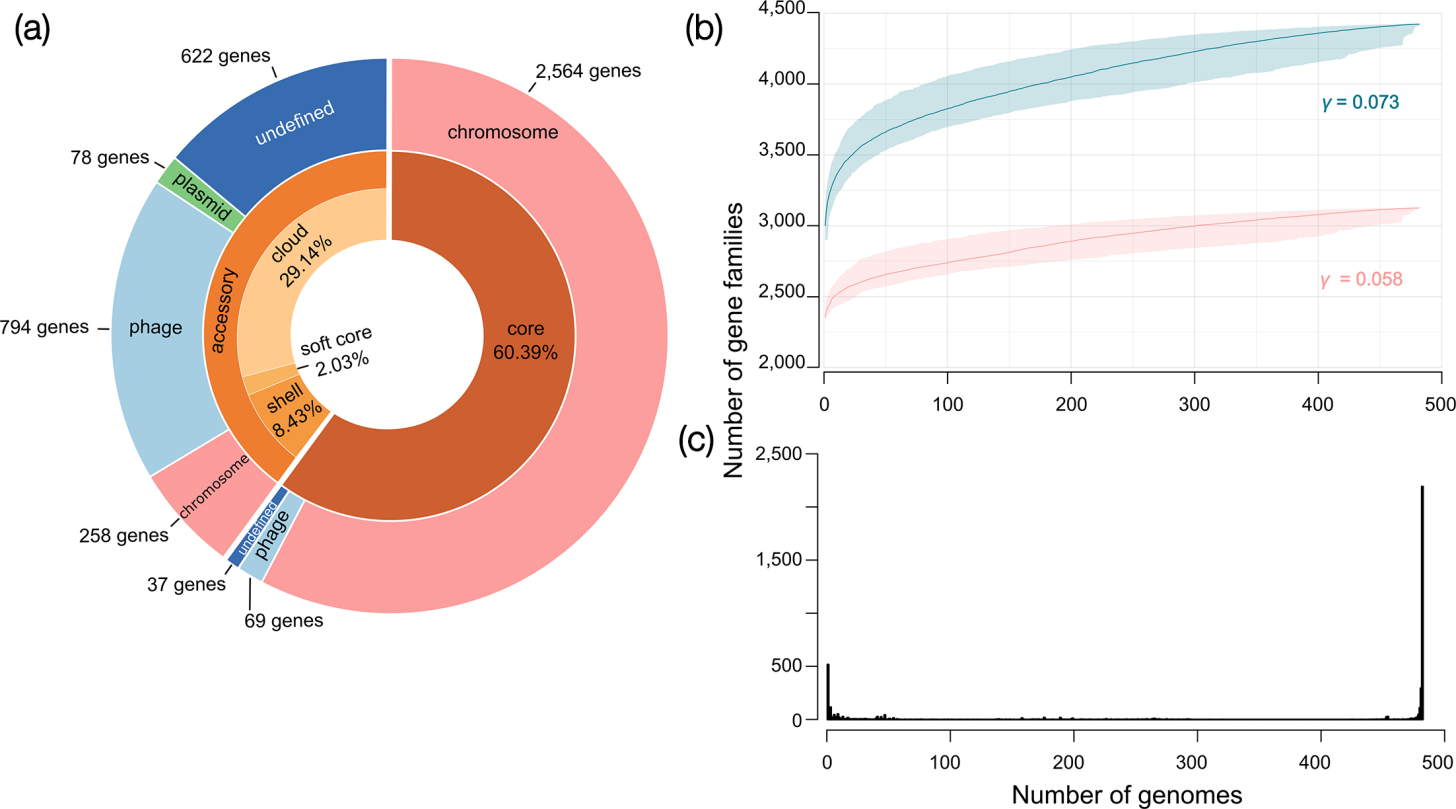
Pangenome composition of ST121 *Listeria monocytogenes*. (a) Core and accessory gene distribution. The inner circle represents the distribution of core (brown) and accessory (orange) genes within the *Listeria monocytogenes* pangenome, derived from 482 genomes. The outer circle further categorizes these genes as chromosomal, phage-derived, plasmid-associated or undefined and specifies their presence within the core or the accessory genome. (b) A rarefaction curve demonstrates the accumulation of pan genes across random combinations of strains. The curve is fitted to the median values of 1,000 random permutations, with shaded areas indicating 95% CIs. The legend displays the power law function parameter ‘*γ*’ at 0.073 for the total pangenome (blue line), indicating an open pangenome, where new genes are continuously added as more genomes are sequenced. The pink line corresponds to the pangenome excluding phage-associated genes. (c) Gene frequency distribution illustrates how genes are shared across varying numbers of genomes.

The distribution of core and accessory genes across the ST121 population was assessed to identify genetic determinants associated with distinct clades or subclades that may have important functional roles. Amongst the 482 genomes, a pangenome of 4,423 genes was identified, including 2,822 chromosomal genes, 863 phage genes, 79 plasmid genes and 659 undefined genes (Fig. 2a). The openness or closedness of the ST121 dataset pangenome was evaluated by constructing a rarefaction curve and fitting the data according to a power law [70]. We computed a *γ* value of 0.073, suggesting that the pangenome of the *Listeria monocytogenes* ST121 dataset is open (Fig. 2b). When phage-associated genes were excluded, the *γ* value decreased to 0.058 (Fig. 2b), reflecting a reduced rate of gene accumulation. While prophages are a major driver of accessory genome diversity, additional factors also contribute to the expansion (openness) of the ST121 pangenome.

Genes present in ≥99% of isolates (i.e. in at least 478 genomes) formed part of the core genome, comprising 2,671 genes (60.39% of the whole pangenome), classified into 2,564 chromosomal, 69 phage, 1 plasmid and 37 undefined genes (Fig. 2 and Table S2). Most undefined core genes were identified as allelic or truncated versions of the reference genes.

Core chromosomal genes in the available schemes from the BIGSdb-Lm database were mapped across the ST121 collection. This analysis identified all genes within the *sigB* operon (*lmo0889*, *lmo0890*, *lmo0891*, *lmo0892*, *lmo0893*, *lmo0894*, *lmo0895* and *lmo0896*), 42 motility-associated genes, 47 virulence-associated genes and all genes from the rhamnose operon (*gtcA*, *lmo1079*, *lmo1080*, *lmo1081*, *lmo1082*, *lmo1083*, *lmo1084* and *lmo2550*) as part of the core genome. Additionally, two genes from the *Listeria* genomic island 2 (LGI-2_LMOSA2310, LGI-2_LMOSA2320), one metal resistance gene (*cadA*) and five antibiotic resistance genes (*fosX*, *lmo0919*, *lmo1695*, *norB* and *sul*) were also core components (Fig. S2). Amongst the virulence-associated genes, *inlA* (*lmo0433*) was identified as a non-core gene, and analysis revealed that all 482 genomes exhibited premature stop codons or gene disruptions, confirming the high prevalence of truncated *inlA* variants in ST121 (Fig. S2).

The phage core genes identified in ST121 were mapped to the complete genome of 757923 and located in 5 dispersed regions (Lm-ST121-01 to 05), each containing at least 11 phage-like genes (11–21 genes). Of these phage-like regions, Lm-ST121-01 closely resembles the monocin gene cluster described in the strain F6854 [89] (Fig. S3). The remaining four phage-like regions showed no matches against known viruses in both INPHARED [75] and the nt collection databases (NCBI database). All 5 regions were identified across more than 20 *Listeria monocytogenes* STs and in at least 3 other *Listeria* species. Searching outside of the genus *Listeria*, only Lm-ST121-01 was observed in the NCBI database, in the uncultivated phage sequence ‘*Caudoviricetes* sp. isolate ctsQg13’ (GenBank: BK030578) from a healthy human stool metagenome [90] (Table S3).

**Fig. 3.**
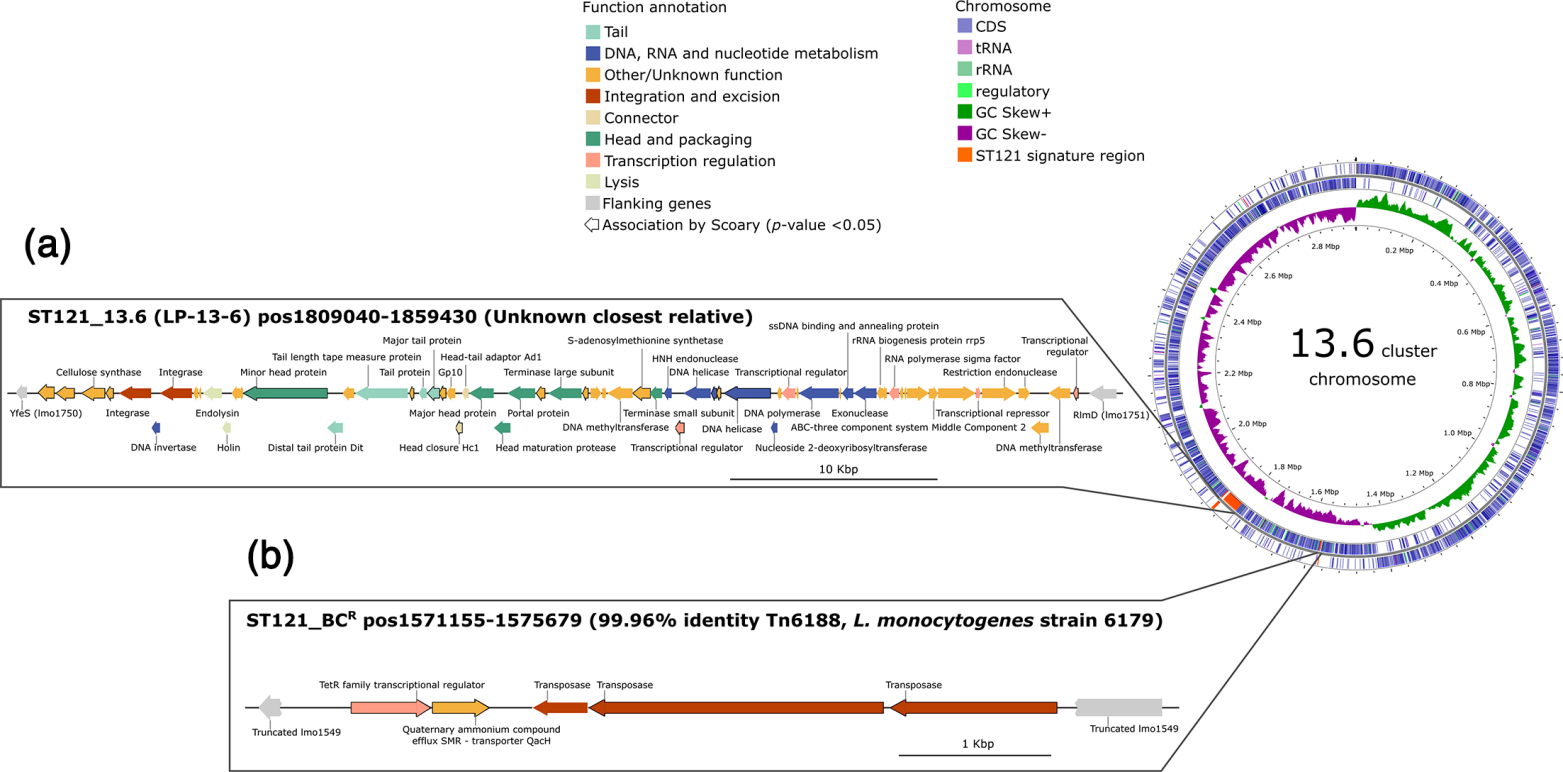
Organization and genomic localization of signature regions associated with clade 13.6 and BC resistance. Gene mapping of pangenome-wide associated traits specific to (a) clade 13.6 and (b) resistance to BC, where associated genes are outlined in black and zoomed in from genomic maps. The position within the genome and nt identity to the closest relative are indicated. Genes are colour coded by function. Scale bars represent gene size. The phage-like signature region associated with clade 13.6 was re-annotated using Pharokka [78], while the genes associated with BC resistance retain their original annotations from Bakta [67].

The ST121 accessory genome (defined here as genes present in 477 genomes or fewer) comprised 1,752 total genes, averaging 330±76 genes per genome. The accessory genes were classified into 258 chromosomal, 794 phage, 78 plasmid and 622 undefined genes (Fig. 2a and Table S2) and mapped onto the dated phylogeny (Fig. S1). Notably, a single plasmid was present in 94.19% of the ST121 collection, exhibiting 99.99% sequence identity with pLM6179 (blastn, 98% query cover), a plasmid frequently found in ST121 genomes [10,14].

Pan-GWASs were conducted to identify genetic regions associated with specific hierarchical clades and with isolates resistant to BC, collectively referred to as ‘signature regions’ (Table S4). In total, seven distinct signature regions of contiguous gene clusters were associated with individual hierarchical clades. These regions were distributed across clades 1.4 (one signature region) (Fig. S4A), 8.1 (two signature regions) (Fig. S4B), 11.4 (two signature regions) (Fig. S4C), 12.2 (one signature region) (Fig. S4D) and 13.6 (one signature region, also present in the closely related clade 13.5) (Fig. 3a). Most of the signature regions were putative prophages, with the exception of a genomic island in clade 11.4 (Table 1). Notably, ten clades (1.5, 7.1, 7.2, 8.3, 8.4, 9.3, 11.3, 12.1, 13.3 and 13.5) yielded no gene associations.

**Fig. 4.**
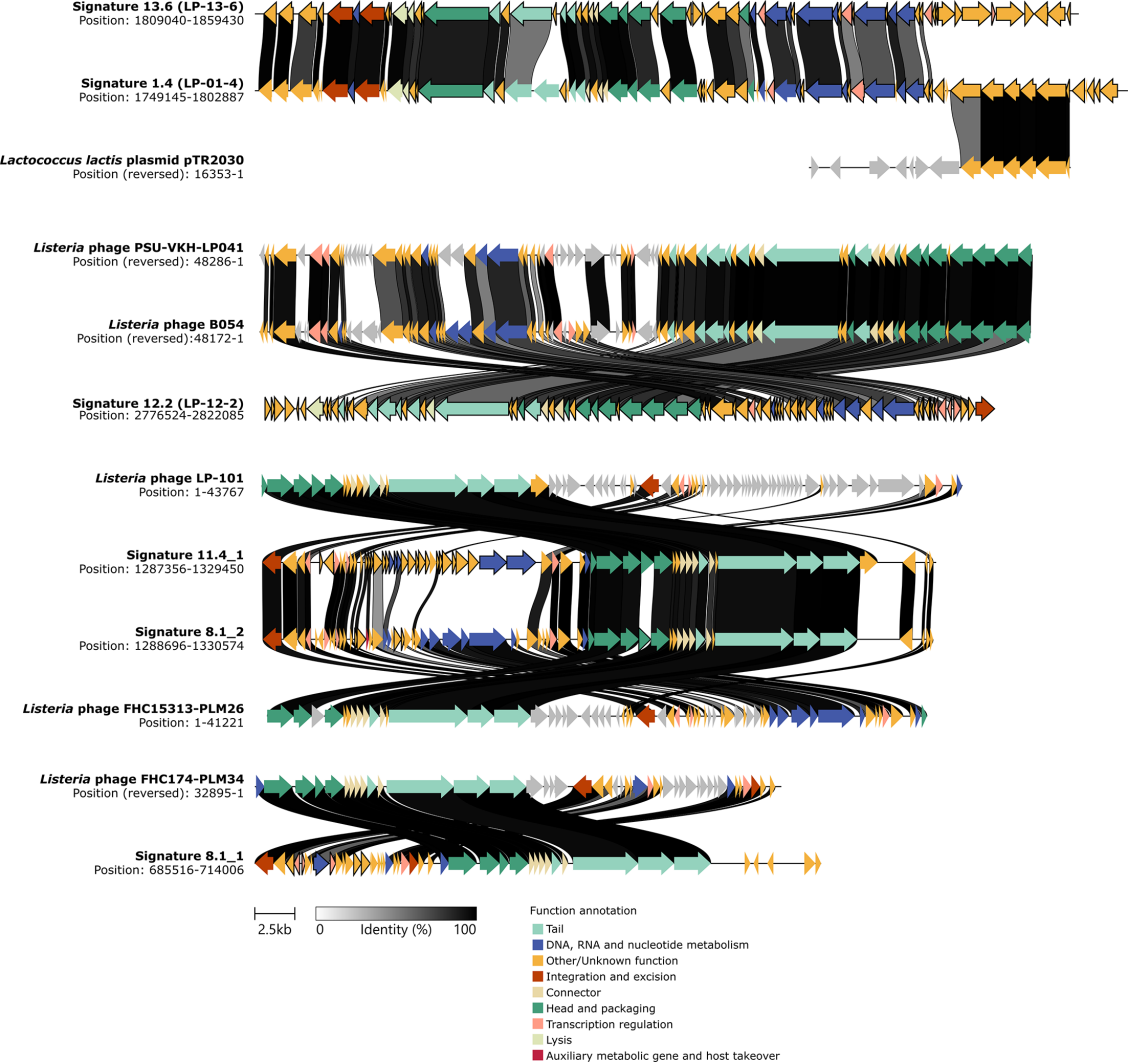
Homology between phage-like genetic signature regions and closest relatives. The relatedness between genetic signature regions and their closest homologous relatives is illustrated through links connecting genes with sequence identity above 50%. Genes are represented as arrows, colour coded by function, with those specifically associated with clades outlined in black. Genes from the closest relatives that do not exhibit homology with the genetic signature regions are displayed in grey. The gradient bar represents nt identity, ranging from 0% (white) to 100% (black). The scale bar indicates gene size.

Putative prophage signature regions exhibited a high level of homology with known *Listeria* phages, except for Signature_13.6 and Signature_1.4 (Fig. 4). The signature region associated with clade 13.6, which includes isolates from Company X [16], had no match with the isolated phage genomes in the INPHARED database. blastn analysis of Signature_13.6 against the nt collection database identified the presence of this region in bacterial genomes, specifically *Listeria monocytogenes* ST3, ST5 and ST204 isolates at 77% coverage with 97.9% identity (Table S5), which has not been previously described. Further investigation of Signature_13.6 showed that it contains all the necessary genes for a complete phage genome, which we have named LP-13–6, along with an additional cluster of genes located upstream of the integrated sequence (Fig. 3a). A putative 14 bp *attL* site was identified adjacent to the integrase gene, indicating that the eight genes upstream of the LP-13-6 genome do not strictly belong to the prophage genome. This additional region includes a gene encoding for cellulose synthase (Fig. 3a).

Based on high genetic similarity (Fig. 4), we propose an evolutionary relationship between LP-13-6 and Signature_1.4, hereafter referred to as LP-01-4. Both regions may also contain phage defence mechanisms, as no additional prophages were predicted in the genomes comprising these clades (Fig. S1). LP-01-4 carries the LlaI restriction-modification (R-M) system (Figs 4 and S4A), previously characterized in the *Lactococcus lactis* conjugative plasmid pTR2030 [91,92]. In contrast, LP-13-6 contains a unique gene cluster at the right end of the integrated sequence, including two putative restriction endonucleases, two methyltransferases and a transcriptional regulator (Fig. 3a), suggesting the presence of a type II R-M system [93,94]. Notably, isolates from clades 13.3 and 7.2, which, despite also carrying this gene cluster, contain additional prophages, exhibit mutations primarily in the transcriptional regulator (Fig. S5), suggesting a potential role in regulating the R-M system.

Signature_12.2, a unique prophage of clade 12.2, here designated as LP-12-2, shares homology with *Listeria* phages PSU-VKH-LP041 and B054 (Table 1 and Fig. S4D). LP-12-2 contains all the essential genes for a complete phage genome, along with a cluster of six genes of unknown function located upstream of the genome, absent in its closest phage relatives (Figs 4 and S4D). These genes are likely part of the prophage, as suggested by the putative 50 bp *attL* site located 390 bp upstream of the first ORF. Similar to clade 12.2, clades 8.1 and 11.4 consist solely of isolates from the UK. The genes associated with clade 8.1 are sparse and distributed across two putative prophages: Signature_8.1_1 and Signature_8.2_2 (Table 1 and Fig. S4B). These prophages are closely related to *Listeria* phages FHC174-PLM34 and FHC15313-PLM26, respectively (Fig. 4), although the genes associated with clade 8.2 exhibit low homology (<50% sequence identity) to their closest relatives. In contrast, genes associated with clade 11.4 are dispersed across two genomic regions: one prophage, Signature_11.4_1, closely related to *Listeria* phage LP-101, and a putative genomic island, Signature_11.4_2, here referred to as LGI-4, which has no known homologues and an unknown function (Table 1 and Fig. S4C). LGI-4 consists of eight coding sequences, including two integrases flanking a serine/threonine protein kinase (STPK), three hypothetical coding sequences and two transcriptional regulators. In addition, genes unique to clade 11.4 and part of Signature_11.4_1 are mostly annotated as hypothetical proteins. However, some identified genes encoding for the V-type ATP synthase subunit F, phenylalanyl-tRNA synthetase beta subunit, replication initiation protein and YopX family protein (Fig. S4C) suggest a potential role in optimizing resource allocation, enhancing host control and modulating host defences [95,97].

BC resistance was associated with a 4.5 kbp genomic region, designated as Signature_BC^R^ (Fig. 3b), present in 92.3% of our ST121 collection. This region corresponds to the transposon Tn6188 (blastn, 99.96% nt identity and 100% query cover to *Listeria monocytogenes* strain 6179), which contains the *qacH* gene known for conferring resistance to quaternary ammonium compounds [27] (Fig. 3b). To investigate the temporal dynamics of genetic adaptation in ST121 to BC exposure, we estimated the chronology of Signature_BC^R^’s introduction. Our analysis indicates two independent introductions of *qacH* into ST121 (Fig. S2). The most recent common ancestor (MRCA) for each subclade where the transposon first appeared was dated to approximately 1973 in clade 1.5 (95% CI 1961–1982) and 1986 in clade 5.1 (95% CI 1968–1999). Additionally, the QacH protein sequence in clade 5.1 exhibits three aa differences compared to clade 1.5 (A60S, A63S and I94L) (Fig. S6).

### Genomic analysis of ST121 *Listeria monocytogenes* complete genomes revealed genetic stability in hotspots

Bacterial hotspots are regions within bacterial genomes highly prone to genetic variation [98]. Because these regions often serve as primary loci for processes such as gene acquisition, recombination or mutation that contribute to adaptation and evolution [98], we investigated variations in 11 hotspots and 3 prophage insertion sites previously defined for *Listeria monocytogenes* [14,29, 32]. This analysis was performed across 16 ST121 complete genomes representing hierarchical clades 1.2, 1.5, 8.4, 9.3, 12.1, 13.5 and 13.6 (Fig. S7). Additionally, we assessed whether the signature regions identified in this study were located within these hotspots (Table 1).

**Fig. 5.**
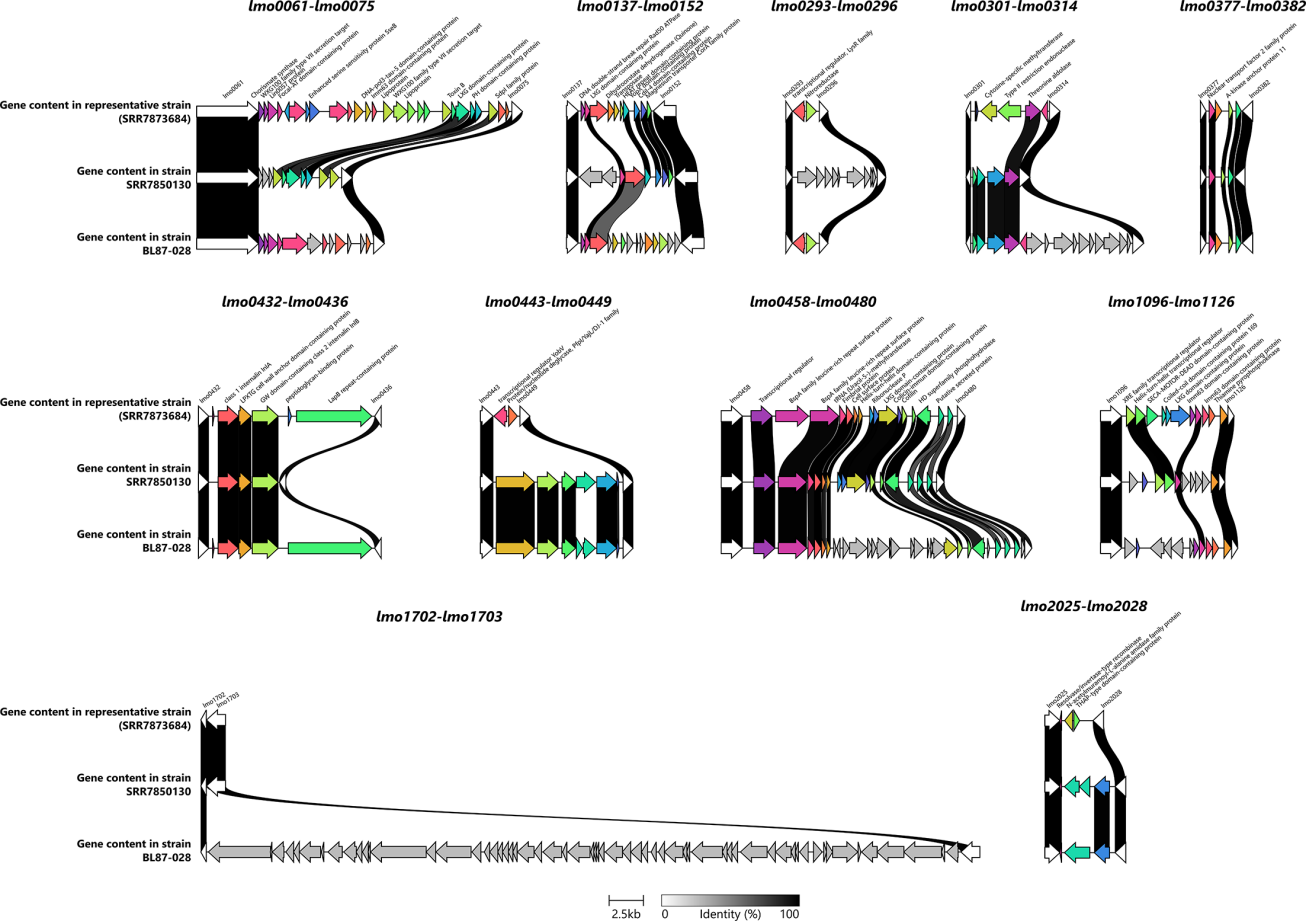
Genetic variation in hotspots across 246242 and BL87-028 genomes. For comparison, the genome of 259390, representing minimal variation in the genetic content of 11 hotspots amongst ST121 isolates, was used to highlight significant differences observed in isolates 246242 and BL87-028. A 50% cutoff was applied to visualize gene connections. Genes were annotated using Bakta [67] and are represented as arrows, with anchor genes shown in white. Genes lacking homology in the compared regions are displayed in grey. The gradient bar indicates nt identity, ranging from 0% (white) to 100% (black), and the scale bar represents gene size.

Overall, the genetic content of the hotspots remained largely conserved amongst the 16 evaluated genomes (Fig. S7), except for isolates 246242 and BL87-028, which exhibited significant differences in most hotspots (Figs 5 and S7A–K). These differences included the loss of single or multiple coding sequences, mostly in strain 246242, and the acquisition of gene clusters, including a prophage in hotspot *lmo1702-lmo1703* of strain BL87-028 (Figs 5 and S7L). No differences were observed in these two strains’ prophage insertion sites *lmo1750-lmo1751*, *lmo1263* and *lmo0271-lmo0272* (Fig. S7M–O). The *lmo0271/lmo0272* prophage insertion site did not contain any genes in the 16 genomes evaluated.

Conversely, at the *lmo1263* prophage insertion site, we identified a prophage in strains 163381 (human origin) and 754322 (environmental origin) (Fig. S7N). Both prophages were closely related to *Listeria* phage B054 and displayed significant sequence variation compared to LP-12-2 (Fig. S7P), which also shares *Listeria* phage B054 as its closest relative.

Of the signature regions identified in this study, only one, LP-01-4, was located within a hotspot, specifically at the *lmo1702/lmo1703* hotspot (Table 1). In contrast, prophage LP-13-6 was present in the previously described prophage insertion site *lmo1750/lmo1751* [14]. All remaining prophage signature regions were integrated at tRNA sites: tRNA-Ser-CGA (Signature 8.1_1), tRNA-Arg-TCT (Signatures 8.1_2 and 11.4_1) and tRNA-Thr-GGT (LP-12–2) (Table 1). Signature 11.4_2, which carries a genomic island, was also inserted at tRNA-Leu-GAG, while Signature_ST121_BC^R^ was inserted into *lmo1549*, which encodes the DNA repair protein RadC.

## Discussion

Understanding the population structure and genetics of *Listeria monocytogenes* is crucial for epidemiological and outbreak investigations, tracking the emergence and presence of antimicrobial resistance and the overall understanding of bacterial evolution and adaptation to different environments. These insights can contribute to developing effective pathogen control strategies when contextualized with epidemiologic and phenotypic characterizations, such as the persistence of biocide tolerance in FPEs.

Amongst *Listeria monocytogenes*, CC121 (largely composed of ST121) is the most prevalent clone found in food isolates [2,11, 99,102]. Our analysis estimates that the most recent common ancestor of *Listeria monocytogenes* ST121, and the root of clade 1, originated around the year 1912 (95% CI 1885–1934). This clade includes genomes from South America and Europe, and the overall widespread distribution of ST121 suggests uncharacterized common reservoirs in food supply chains that were subject to global trade. Similar studies have drawn historical correlations between the global spread of *Listeria monocytogenes* CC1 and events such as the transatlantic livestock trade and the expansion of cattle farming and food industrialization [103]. The successful expansion of ST121 clades across multiple countries is notable, particularly with the fixation of the transposon Tn6188. This transposon harbours two variants of the *qacH* gene between clade 1.5 and other clades, potentially contributing to their expansion. The lower G+C content of Tn6188 suggests a relatively recent acquisition by *Listeria monocytogenes* [27]. We estimate that Tn6188 was first transferred into ST121 in 1973 in clade 1.5, with another variant introduced in clade 5.1 around 1986.

Pangenome analysis provided insights into the distribution of core and accessory genes within the ST121 population. We observed an open pangenome but stable genomic backbone, with 2,671 core genes, representing ~89% of the genome coding sequences. This is consistent with previous studies reporting a 75% core genome in *Listeria monocytogenes*, highlighting the species’ relatively low genetic variability [104]. Amongst the core genes, 69 were identified as phage-borne, with prophage-like remnants in over 20 *Listeria monocytogenes* STs and at least 3 other *Listeria* species, indicating a long-standing relationship between *Listeria* and prophages. One prophage-like cluster, Lm-ST121-01, shows close homology to the monocin gene cluster in strain F6854 [105]. Monocins, resembling both high-molecular-weight bacteriocins and phage tails (tailocins), exhibit lytic activity targeting genus-specific bacteria but do not cause productive infection [105,106]. Monocins are expressed during intracellular growth across lineages I, II and III [107] and may provide a competitive advantage in host colonization by modulating the intestinal microbiome [89,106]. The ancestral connection between monocins, prophages and lytic activity prompts further questions about their role in interactions between *Listeria monocytogenes*, its hosts and the microbiome.

The open pangenome of ST121 appears to be significantly influenced by the acquisition of phage-related genes, with pan-GWAS analysis identifying seven signature regions, mostly prophages, unique to specific clades. Notably, these prophage-associated regions were not restricted to ST121, suggesting a broader network of horizontal gene transfer across *Listeria* genotypes (Table S5). This distribution further supports the idea that ST121 shares mobile genetic elements with other STs, likely due to phage-mediated processes in common ecological niches. One prophage, LP-13-6, was identified in both clade 13.6 and the closely related clade 13.5, suggesting a common evolutionary origin. Whereas clade 13.6 is associated with sandwiches, salads and a specific manufacturing environment (Company X), clade 13.5 exhibits distinct epidemiological characteristics, with isolates found in Estonia, Poland and France and clinical cases in the UK. In Estonia, isolates were primarily sourced from fish, while in Poland, they were isolated from meat, reflecting a broader food-related distribution across different regions. The epidemiological divergence may reflect adaptation to different environments or production settings, with both clades maintaining the LP-13-6 prophage.

A notable feature of isolates from clade 13.6 is the putative cellulose synthase cluster, present outside the *attL* boundary of LP-13–6, which suggests a specialized transduction event from an unknown donor strain into the ancestral 13.5/13.6 strain. We hypothesize the cellulose synthase gene to contribute to biofilm production [108] aligning with the persistence of clade 13.6 isolates in the Company X environment. In this setting, the microbiota is dominated by *Pseudomonas fluorescens* and other taxa, such as *Sphingomonas aerolata*, while *Listeria* occurs at low abundance and has adapted to this specific niche, potentially benefiting from mutualistic interaction within the microbial community [16,40]. Additionally, the putative type II R-M system found in LP-13-6, along with the cellulose synthase cluster, could provide survival and adaptation advantages by protecting *Listeria monocytogenes* from other phages [109]. This mirrors the hypothesis proposed for ST121 strain AB27, where the absence of additional prophages is explained by the presence of the LlaI R-M system [14]. In our study, clade 1.4 strains carrying LP-01–4 and the LlaI R-M system, essential for restriction endonuclease activity and conferring phage resistance [91,92], also lacked additional prophages.

We estimate that clade 13.6 emerged around 1997 (95% CI 1990–2002), earlier than the previously reported estimate of 2014 [16]. This discrepancy may be due to the exclusion of prophage sequences in core genome-based analyses, which can otherwise distort phylogenetic signals and MRCA dating by incorporating multiple genes from a single event [32,110]. Alternatively, the earlier date may represent the true timing of the strain’s introduction into the manufacturing environment, prior to enhanced surveillance efforts.

We also identified a unique prophage, LP-12-2, associated with clade 12.2, closely related to the *Listeria* phages B054, isolated from *Listeria innocua* [111], and PSU-VKH-LP041, recovered from *Listeria monocytogenes* in a seafood processing environment [112]. These phages are known for their genomic synteny [113,114], although LP-12-2 differs by a cluster of six genes of unknown function. The emergence of clade 12.2 around 2005, its first isolation in 2014 and the growing number of isolates since 2021 warrant further investigation into the functional role of LP-12-2.

Comparative analyses revealed an evolutionary relationship between the prophage in clade 11.4 (Signature_11.4_1) and its closest relative, LP-101, based on the conservation of structural genes. Differences in replication and accessory genes, including those encoding V-type ATP synthase subunit F, phenylalanyl-tRNA synthetase beta subunit, replication initiation protein and YopX family protein (associated with virulence [96]), may offer selective advantages. ATP synthases help maintain ion gradients and optimize energy efficiency [97], while the structural similarity of V-type ATP synthase subunit F to the *Escherichia coli* chemotaxis response regulator CheY [115] suggests a potential role in modulating bacterial stress response [116]. Phenylalanyl-tRNA synthetase is essential for accurate translation under oxidative stress [117] and may be used by the phage [95] to ensure efficient protein synthesis under stressful conditions.

We also identified a novel genomic island in clade 11.4, LGI-4, carrying an STPK. STPKs are known to regulate cellular functions, including virulence, biofilm formation and stress response in various pathogens, such as *Streptococcus* spp., *Yersinia pseudotuberculosis* and *Mycobacterium tuberculosis* [118,123]. In *Listeria monocytogenes,* the STPK PrkA is essential for cell wall resistance, intracellular survival and virulence [124]. This suggests that LGI-4 may contribute to both environmental resilience and pathogenic potential.

Finally, previous pangenome analysis suggests that accessory genes tend to accumulate in hyper-variable hotspots [32]. However, in our study, we observed that these hotspots, along with two additional hotspots (*lmo0443-lmo0449* and *lmo1702-lmo1703*) [14,29], remained stable across isolates, except for isolates BL87-028 and 246242. Notably, 246242 had 2,768 coding sequences, 233 fewer than the average across the ST121 genome panel. This reduction represents ~7.8% fewer coding sequences, suggesting bacterial streamlining driven by selection for the removal of non-essential genetic elements [125], leading to a reduced genome size.

## Conclusion

We provided a detailed genomic portrait of *Listeria monocytogenes* ST121, offering insights into its evolutionary history in diverse environmental and food niches and the potential mechanisms driving its persistence in those niches. The analysis of the pangenome of ST121 provided insights into the distribution of core and accessory genes, revealing a highly stable core backbone but with an open pangenome where a significant proportion of accessory genes was phage derived, suggesting their potential roles of phage-driven diversity in adaptation and survival within specific environments. The presence of one signature region, phage LP-13–6, suggests a compelling hypothesis for the durable success of clade 13.6 within an FPE, potentially contributing to biofilm formation within a community of other environmental microbiota present in the facility. Overall, our findings highlight the importance of understanding the population structure and genetics of *Listeria monocytogenes* for epidemiological surveillance, antimicrobial resistance tracking, food safety and public health interventions. Further research into the evolutionary dynamics and functional roles of prophages and LGI-4 within *Listeria monocytogenes* populations is needed to enhance our comprehension of its pathogenicity and ecological adaptability.

## Supplementary material

10.1099/mgen.0.001397 Uncited Supplementary Material 1.

10.1099/mgen.0.001397 Uncited Supplementary Material 2.

## Abbreviations

BAPS: bayesian analysis of population structure
blast: Basic Local Alignment Search Tool
CC: clonal complex
CI: confidence interval
FPE: food production environment
INPHARED: Infrastructure for a Phage Reference Database
LHB-MHA: lysed horse blood-Mueller–Hinton agar
MIC: minimum inhibitory concentration
MRCA: most recent common ancestor
pan-GWAS: pangenome-wide association study
R-M: restriction-modification
ST: sequence type
