# Supplementary material for: Population structure and gene flux of Listeria monocytogenes ST121 reveal prophages as a candidate driver of adaptation and persistence in food production environments
Source: Microb Genom. 2025 Apr 17;11(4):001397. doi: 10.1099/mgen.0.001397 (PMC12006667; doi:10.1099/mgen.0.001397)
Supplement: Uncited Supplementary Material 1. [file mgen-11-01397-s001.pdf]

Supp Figure 1. Time-calibrated phylogenetic tree and accessory genome of *L. monocytogenes* ST121. Accessory genome gene families were categorised as non-prophage chromosomal (pink), prophage (light blue), plasmid (green), or undefined (dark blue), with gene families present in ≥ 478 strains excluded. Gene presence and absence were visualised using a 90% identity threshold and aligned with the time-calibrated phylogenetic tree shown in Figure 1D, providing an integrated view of the accessory genome's distribution across the evolutionary timeline.

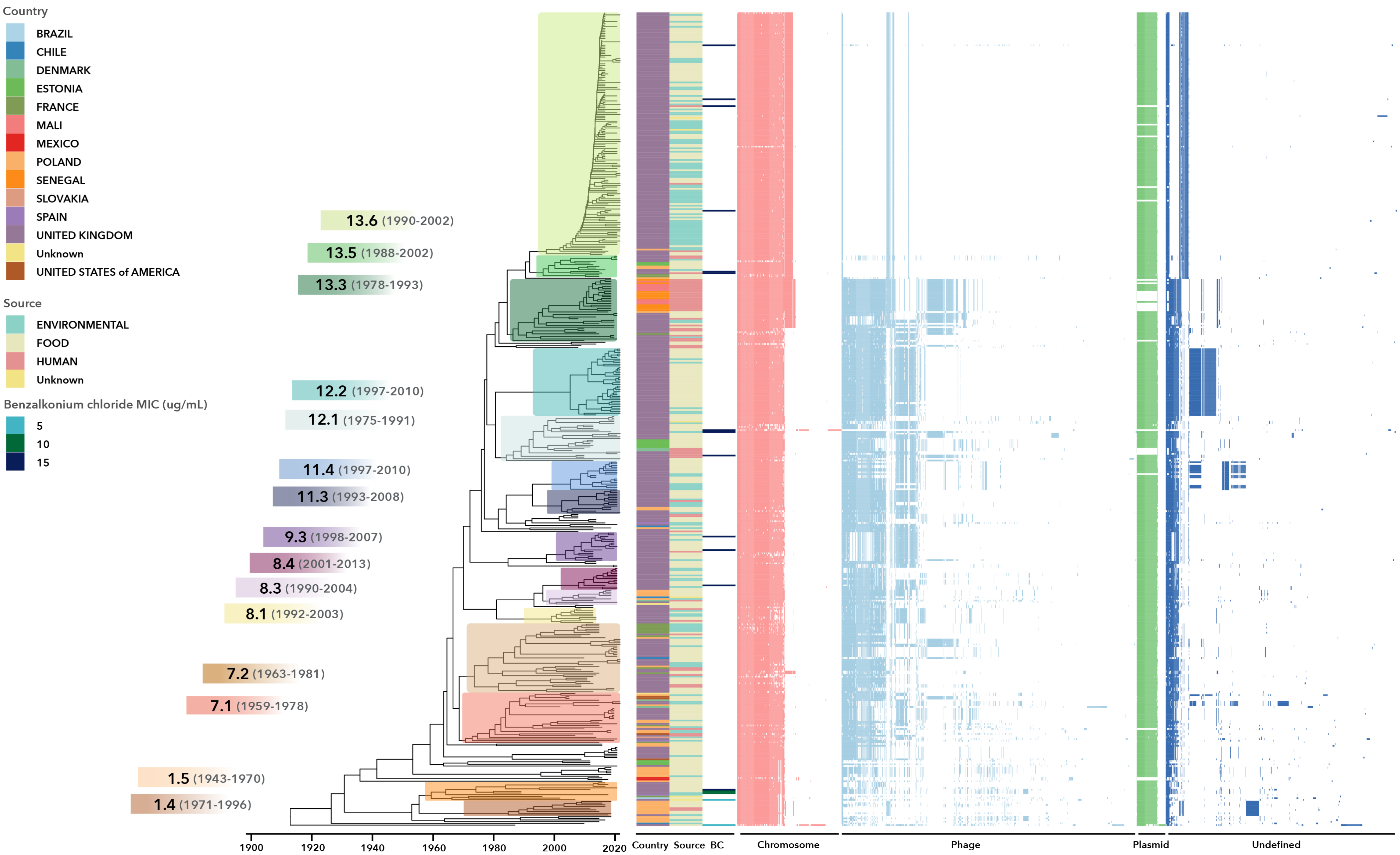

Supp Figure 2. The presence/absence of genes associated with virulence, resistance to metal, disinfectant, antibiotics, stress islands, *Listeria* genomic Islands, motility, rhamnose, and the *sigB* operon. Brown boxes represent the presence of core genes, orange boxes accessory genes, black boxes denote genes with premature stop codons or gene disruption, and grey boxes genes with internal truncation. Acquisition of *qacH* in clades 1.5 and 5.1 denoted with asterisks (*).

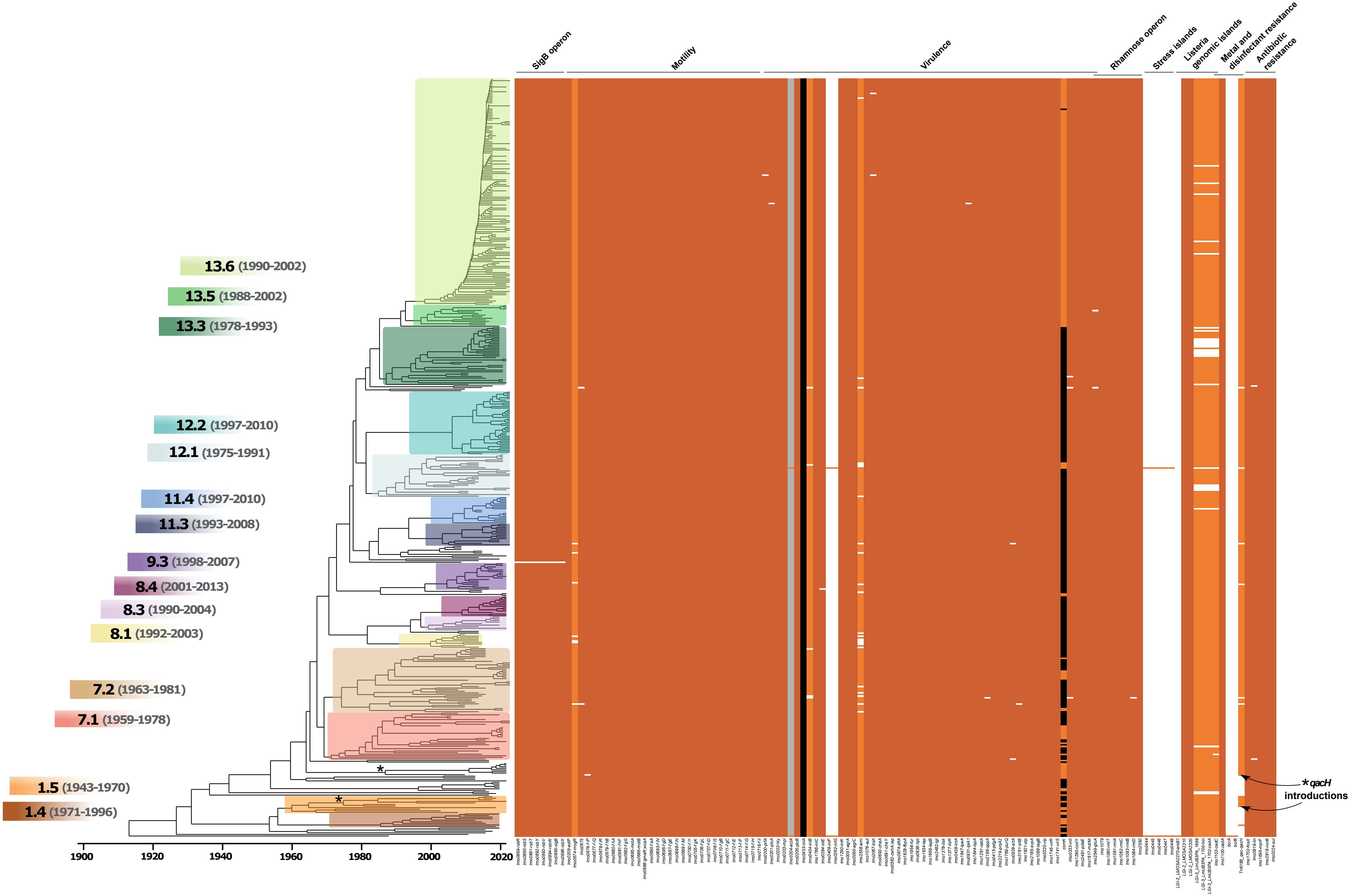

Supp Figure 3. The core genome of ST121 *L. monocytogenes*. A) Genomic map of non-prophage chromosomal and prophage core genes. Genes present in ≥99% of isolates were mapped to the complete genome SRR9298670, while non-coloured areas correspond to accessory genes. B) Phage-associated core genes. 69 genes of phage origin were identified in the core genome, clustered in five different genomic locations (Lm-ST121-01 to 05). Annotations of phage-gene clusters are colour-coded according to function. Lm-ST121-01's close relatedness to the monocin gene cluster (strain F6854) is shown by homology comparison. No close relatives were identified for Lm-ST121-02 to 05. The scale bar represents gene size, and the gradient bar ranges from 0% (white) to 100% (black), indicating nucleotide identity.

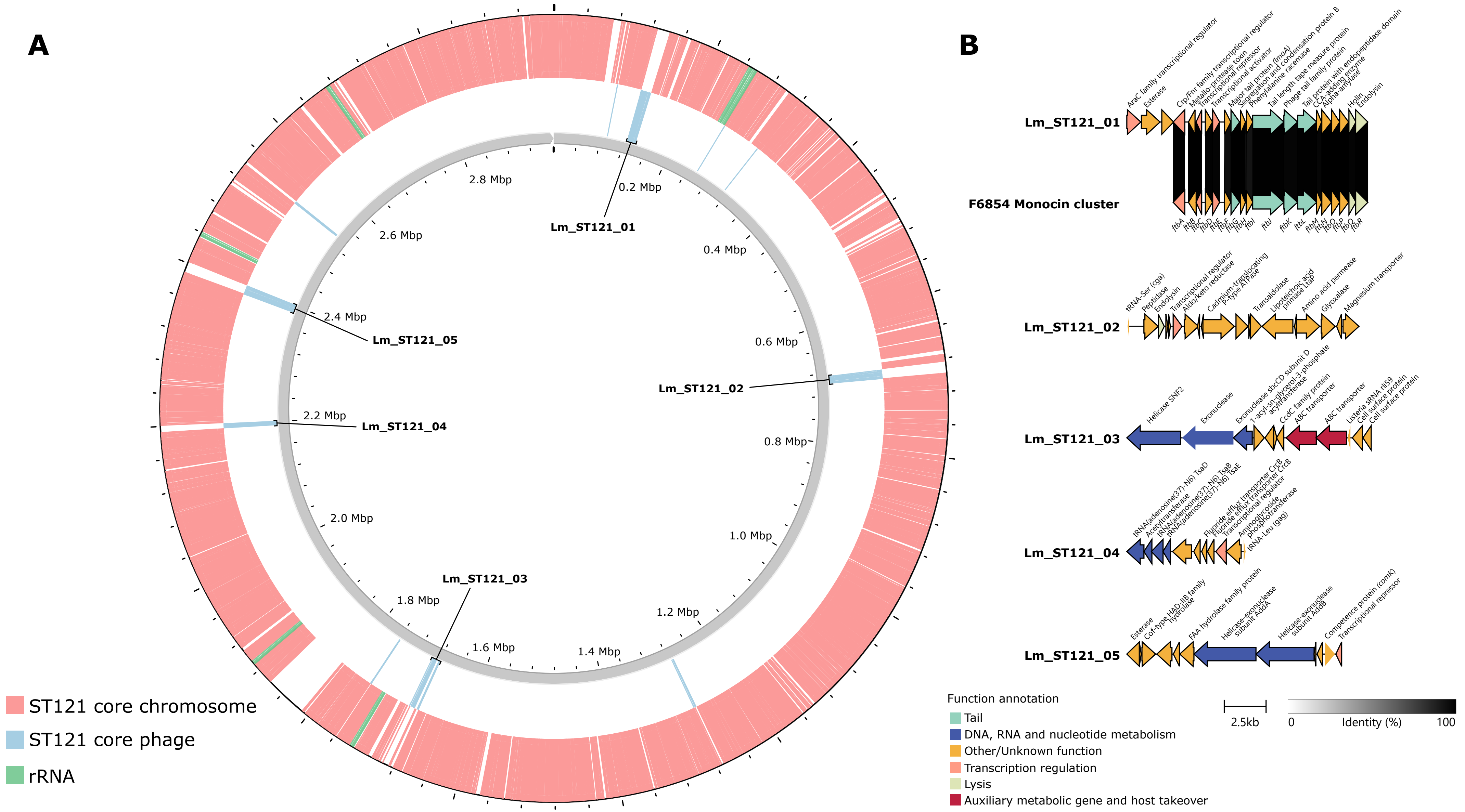

Supp Figure 4. Organisation signature regions associated with hierarchical clades 1.4, 8.1, 11.4, and 12.2. Gene mapping of pangenome-wide associations for clades A) 1.4, B) 8.1, C) 11.4, and D) 12.2, highlighting genes significantly associated with these clades (outlined in black) and zoomed in from the genomic maps. The genomic location and nucleotide identity to the closest relative are indicated. Genes are colour-coded according to function, and scale bars represent gene size. Phage-like signature regions were re-annotated using Pharokka, while the genes within the genomic island (Signature region 11.4_2) retain their original Bakta annotations.

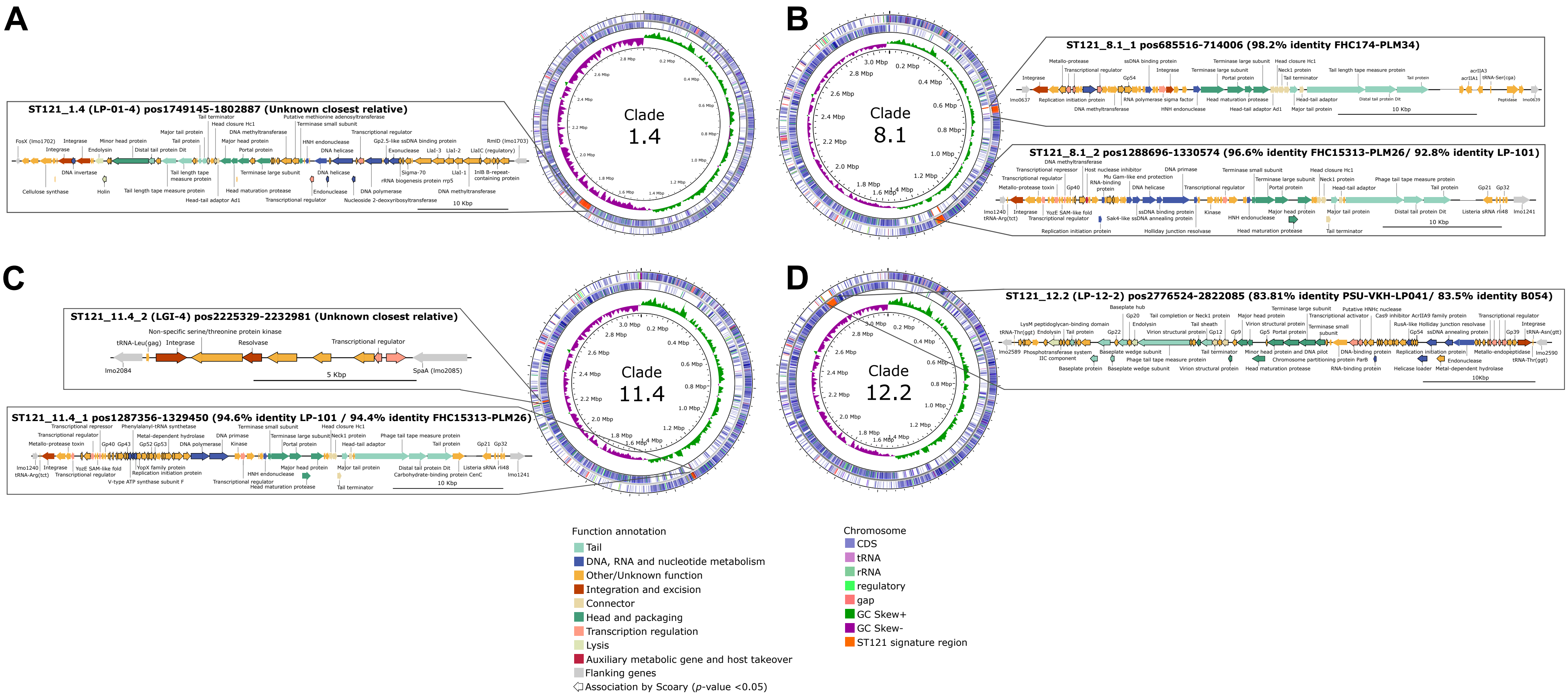

Supp Figure 5. Mutational variations in the putative transcriptional regulator of the restriction-modification system in clades 13.3 and 7.2. Amino acid sequences of the putative transcriptional regulator from ST121 clades 13.3 and 7.2 were aligned using MAFFT, with conserved residues highlighted using Boxshade. The consensus sequence is displayed below each alignment block, where asterisks (*) denote fully conserved positions and dots (.) indicate similar residues. The helix-turn-helix (HTH) sequence motif was identified using the GenomeNet Bioinformatics Tool - MOTIF.

HTH MOTIF

```
Reg_SRR9298670_13.6          1 MDTPTKQKIIDLRNEGVGYKRIADQLDVSVNSVKSFRRSKGLTGNRTKWNAKQALDAAEV
Reg_ 78213_Q0720_13.3        1 MDTPTKQKIIDLRNEGVGYKRIADQLDVSVNSVKSFCRSKGLTGNRTNWNARQALEVAEV
Reg_ 1661_H133200172_7.2     1 MDTPTKQKIIDLRNEGVGYKRIADQLDVSVNSVKSFCRSKGLTGNRTNWNARQALEVAEV
consensus                    1 ************************************.**********.***.***..***

Reg_SRR9298670_13.6         61 TQIKKS
Reg_ 78213_Q0720_13.3       61 THIKKS
Reg_ 1661_H133200172_7.2    61 THIKKS
consensus                   61 *.****
```

Supp Figure 6. Amino acid sequence alignment of QacH from ST121 clades 1.5, 7.1, 12.1 and 13.6. Amino acid sequences of QacH from ST121 clades 1.5, 7.1, 12.1 and 13.6 were aligned using MAFFT. Conserved residues are highlighted using Boxshade, with key functional residues of QacC highlighted in red, following the approach outlined by (98). The consensus sequence is shown below each alignment block, with asterisks (*) indicating fully conserved positions, and dots (.) representing similar residues.

```
QacH_BL89-020_1.5        1 MSYLYLALAIVGEIIGSSLLKASEGFSKLFPTIGVIIAFVVSFFFLSLSLKTIPLNTAYA
QacH_LS12-859_1.5        1 MSYLYLALAIVGEIIGSSLLKASEGFSKLFPTIGVIIAFVVSFFFLSLSLKTIPLNTAYA
QacH_47974_CLIP_7.1      1 MSYLYLALAIVGEIIGSSLLKASEGFSKLFPTIGVIIAFVVSFFFLSLSLKTIPLNTAYS
QacH_SRR6807418_12.1     1 MSYLYLALAIVGEIIGSSLLKASEGFSKLFPTIGVIIAFVVSFFFLSLSLKTIPLNTAYS
QacH_SRR9298670_13.6     1 MSYLYLALAIVGEIIGSSLLKASEGFSKLFPTIGVIIAFVVSFFFLSLSLKTIPLNTAYS
consensus                1 ***********************************************************.

QacH_BL89-020_1.5       61 LWAGLGLVLTTIISVLIWKEKLNMASIAGITLIIAGVVILNLFGPGHGEPDHKASETITI
QacH_LS12-859_1.5       61 LWAGLGLVLTTIISVLIWKEKLNMASIAGITLIIAGVVILNLFGPGHGEPDHKASETITI
QacH_47974_CLIP_7.1     61 LWSGLGLVLTTIISVLIWKEKLNMASIAGITLILAGVVILNLFGPGHGEPDHKASETITI
QacH_SRR6807418_12.1    61 LWSGLGLVLTTIISVLIWKEKLNMASIAGITLILAGVVILNLFGPGHGEPDHKASETITI
QacH_SRR9298670_13.6    61 LWSGLGLVLTTIISVLIWKEKLNMASIAGITLILAGVVILNLFGPGHGEPDHKASETITI
consensus               61 **.******************************.**************************

QacH_BL89-020_1.5      121 NEE
QacH_LS12-859_1.5      121 NEE
QacH_47974_CLIP_7.1    121 NEE
QacH_SRR6807418_12.1   121 NEE
QacH_SRR9298670_13.6   121 NEE
consensus              121 ***
```

Supp Figure 7. Variation in genetic content of hotspots and prophage insertion sites among ST121 isolates. Variations of 11 hotspots A) *lmo0061-lmo0075*, B) *lmo0137-lmo0152*, C) *lmo0293-lmo0296*, D) *lmo0301-lmo0314*, E) *lmo0377-lmo0382,* F) *lmo0432-lmo0436*, G) *lmo0458-lmo0480*, H) *lmo1096-lmo1126*, I) *lmo2025-lmo2028*, J) *lmo0443-lmo0449*, K) *lmo1702-lmo1703*, and three prophage insertion sites, M) *lmo1750-lmo1751*, N) *lmo1263*, O) *lmo0271-lmo0272* across 16 complete genomes of ST121, spanning hierarchical clades 1.2, 1.5, 8.4, 9.3, 12.1, 13.5, and 13.6. P) Comparison between LP-12-2 and putative prophages found in prophage insertion site *lmo1263*. Differences in the genetic content of hotspots were observed in isolates SRR7850130 and BL87-028, with detailed annotations provided in L. A cutoff of 50% sequence identity was applied for visualising gene connections. Genes were annotated using Bakta and depicted as arrows, with anchor genes shown in white. Genes with no homology within the compared genomic area are shaded in grey. The scale bar represents gene size, with the gradient bar ranging from 0% (white) to 100% (black), indicating nucleotide identity.

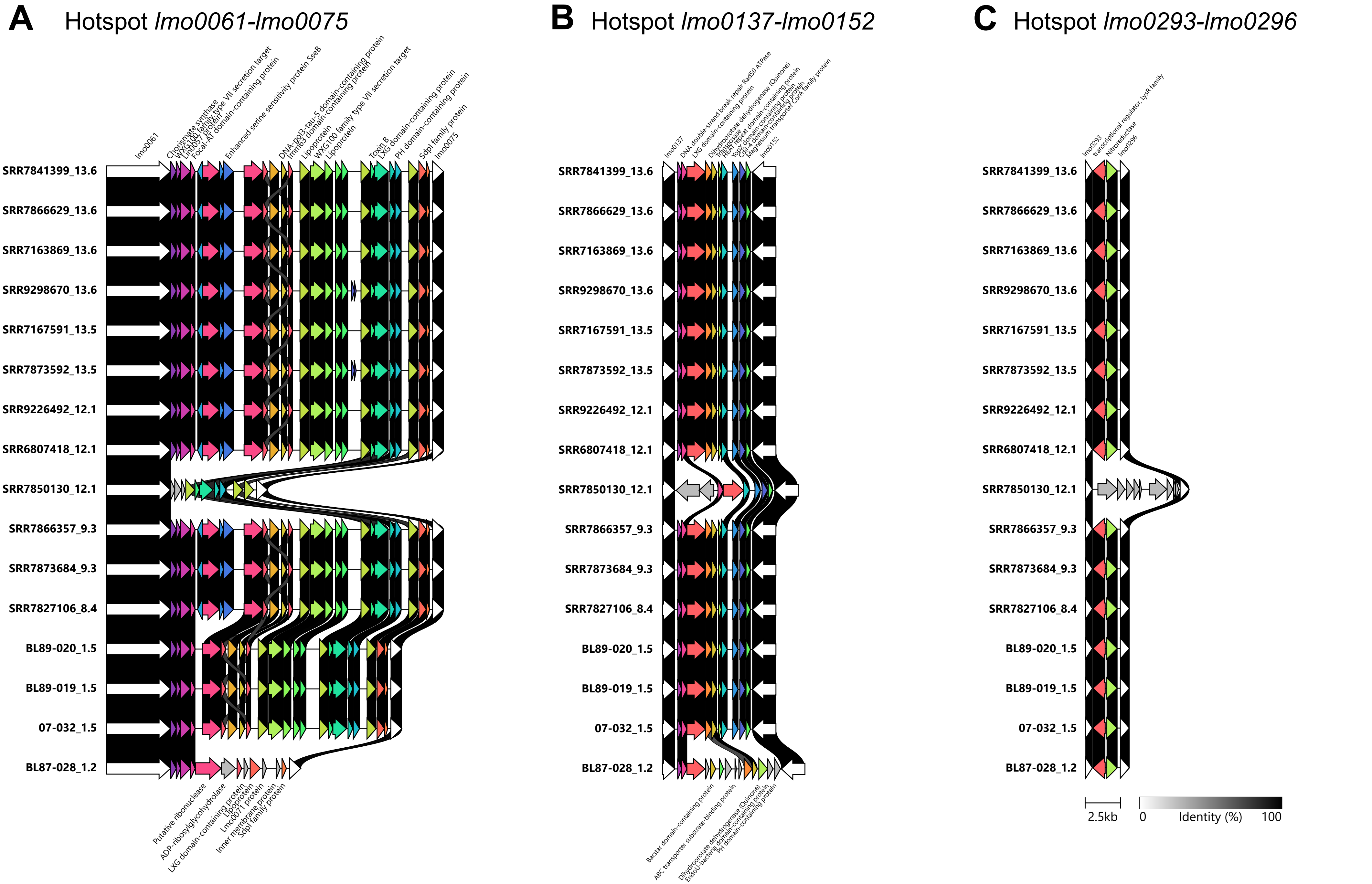

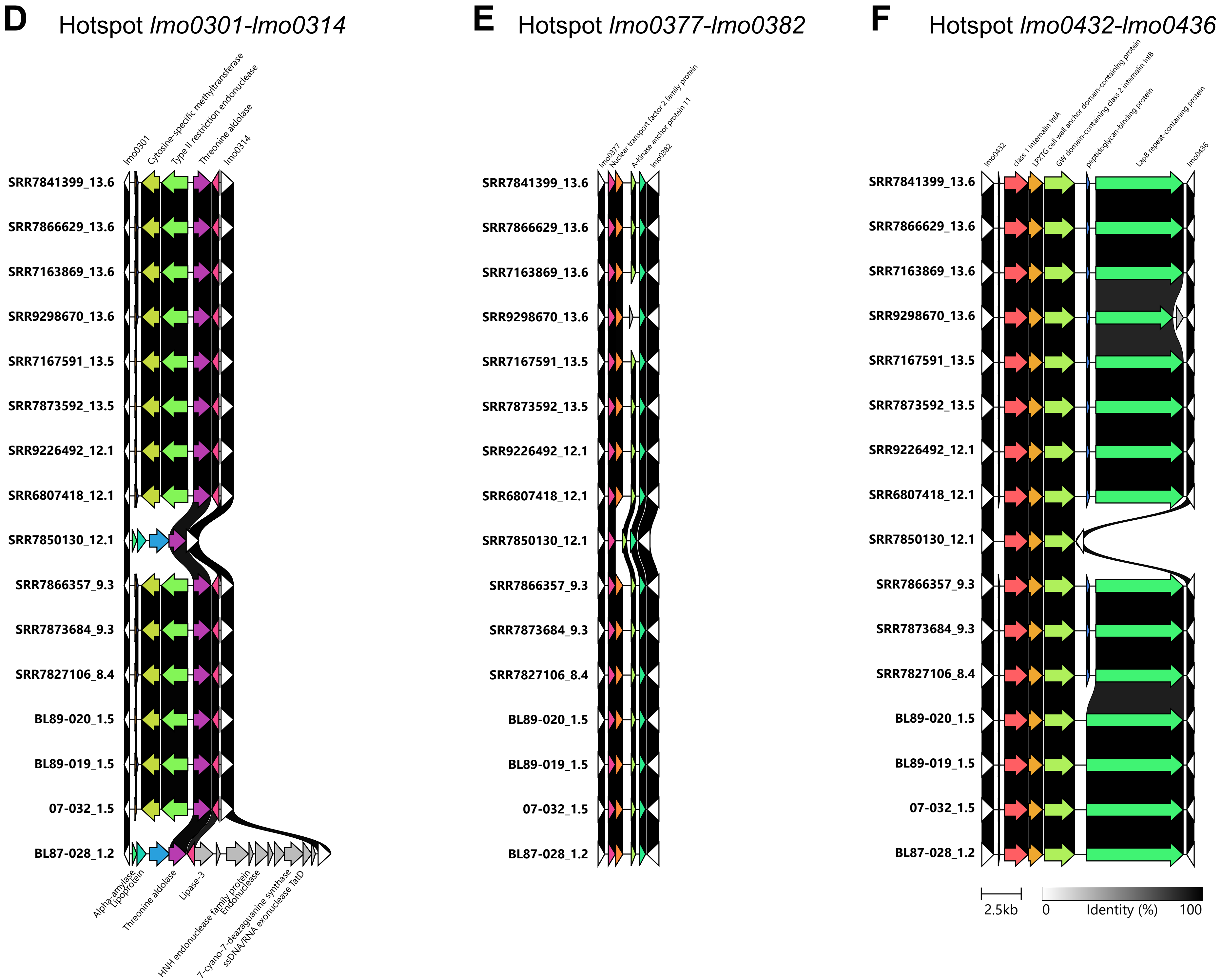
D
Hotspot lmo0301-lmo0314
lmo0301
Cytosine-specific methyltransferase
Type II restriction endonuclease
Threonine aldolase
lmo0314
SRR7841399_13.6
SRR7866629_13.6
SRR7163869_13.6
SRR9298670_13.6
SRR7167591_13.5
SRR7873592_13.5
SRR9226492_12.1
SRR6807418_12.1
SRR7850130_12.1
SRR7866357_9.3
SRR7873684_9.3
SRR7827106_8.4
BL89-020_1.5
BL89-019_1.5
07-032_1.5
BL87-028_1.2
Alpha-amylase
Lipoprotein
Threonine aldolase
Lipase-3
HNH endonuclease family protein
Endonuclease
7-cyano-7-deazaguanine synthase
ssDNA/RNA exonuclease TatD
E
Hotspot lmo0377-lmo0382
lmo0377
Nuclear transport factor 2 family protein
A-kinase anchor protein 11
lmo0382
SRR7841399_13.6
SRR7866629_13.6
SRR7163869_13.6
SRR9298670_13.6
SRR7167591_13.5
SRR7873592_13.5
SRR9226492_12.1
SRR6807418_12.1
SRR7850130_12.1
SRR7866357_9.3
SRR7873684_9.3
SRR7827106_8.4
BL89-020_1.5
BL89-019_1.5
07-032_1.5
BL87-028_1.2
F
Hotspot lmo0432-lmo0436
lmo0432
class 1 internalin InlA
LPXTG cell wall anchor domain-containing protein
GW domain-containing class 2 internalin InlB
peptidoglycan-binding protein
LapB repeat-containing protein
lmo0436
SRR7841399_13.6
SRR7866629_13.6
SRR7163869_13.6
SRR9298670_13.6
SRR7167591_13.5
SRR7873592_13.5
SRR9226492_12.1
SRR6807418_12.1
SRR7850130_12.1
SRR7866357_9.3
SRR7873684_9.3
SRR7827106_8.4
BL89-020_1.5
BL89-019_1.5
07-032_1.5
BL87-028_1.2
2.5kb
0
Identity (%)
100

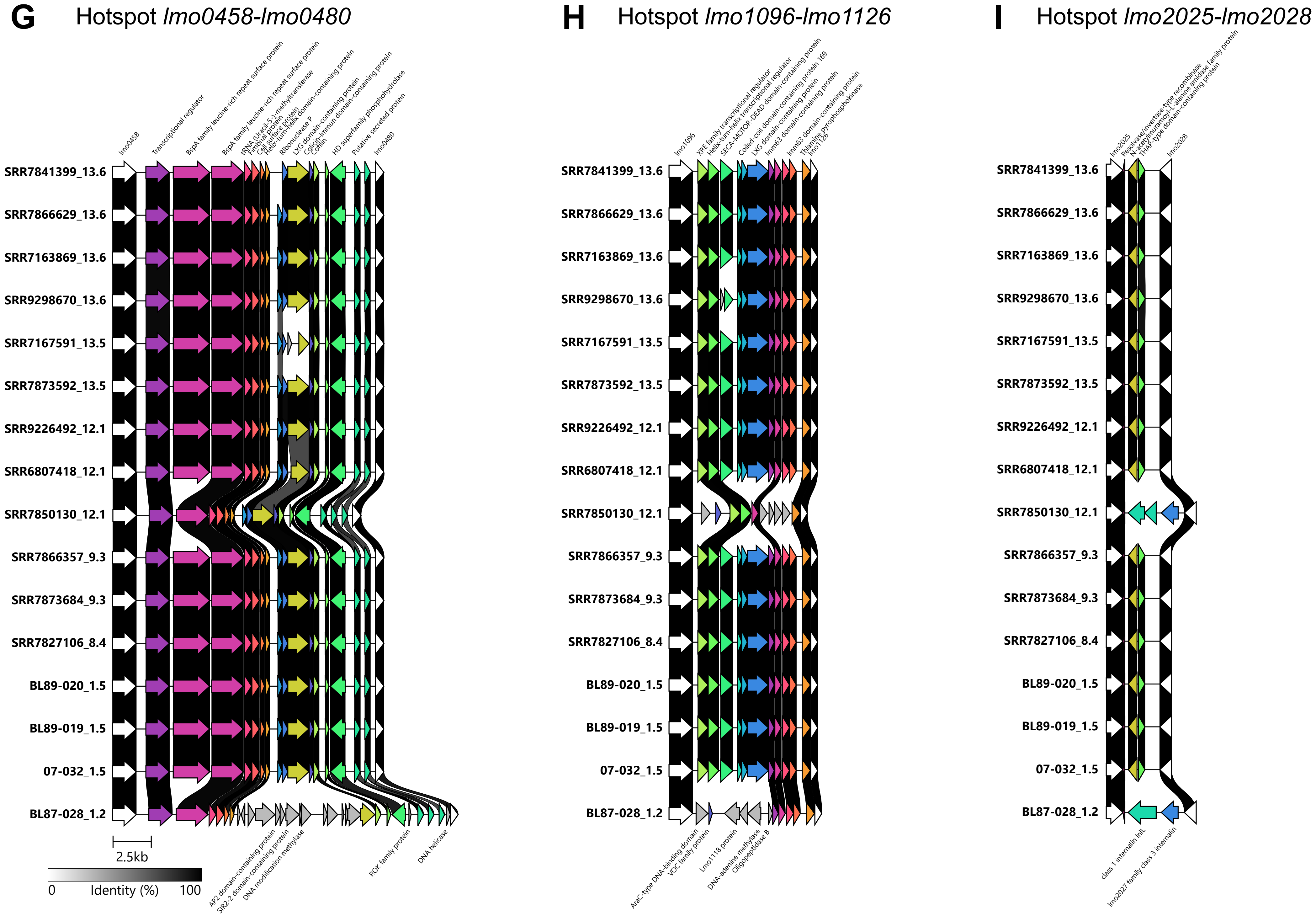
G
Hotspot lmo0458-lmo0480
lmo0458
Transcriptional regulator
BspA family leucine-rich repeat surface protein
BspA family leucine-rich repeat surface protein
tRNA (Uracil-5-)-methyltransferase
Fimbrial protein
Cell surface protein
Helix-turn-helix domain-containing protein
Ribonuclease P
LXG domain-containing protein
Colicin-immun domain-containing protein
Cohlin
HD superfamily phosphohydrolase
Putative secreted protein
lmo0480
SRR7841399_13.6
SRR7866629_13.6
SRR7163869_13.6
SRR9298670_13.6
SRR7167591_13.5
SRR7873592_13.5
SRR9226492_12.1
SRR6807418_12.1
SRR7850130_12.1
SRR7866357_9.3
SRR7873684_9.3
SRR7827106_8.4
BL89-020_1.5
BL89-019_1.5
07-032_1.5
BL87-028_1.2
2.5kb
0
Identity (%)
100
AP2 domain-containing protein
SIR2-2 domain-containing protein
DNA modification methylase
ROK family protein
DNA helicase
H
Hotspot lmo1096-lmo1126
lmo1096
XRE family transcriptional regulator
Helix-turn-helix transcriptional regulator
SECA-MOTOR-DEAD domain-containing protein
Coiled-coil domain-containing protein 169
LXG domain-containing protein
Imm63 domain-containing protein
Imm63 domain-containing protein
Thiamine pyrophosphokinase
lmo1126
SRR7841399_13.6
SRR7866629_13.6
SRR7163869_13.6
SRR9298670_13.6
SRR7167591_13.5
SRR7873592_13.5
SRR9226492_12.1
SRR6807418_12.1
SRR7850130_12.1
SRR7866357_9.3
SRR7873684_9.3
SRR7827106_8.4
BL89-020_1.5
BL89-019_1.5
07-032_1.5
BL87-028_1.2
AraC-type DNA-binding domain
VOC family protein
Lmo1118 protein
DNA-adenine methylase
Oligopeptidase B
I
Hotspot lmo2025-lmo2028
lmo2025
Resolvase/invertase-type recombinase
N-acetylmuramoyl-L-alanine amidase family protein
THAP-type domain-containing protein
lmo2028
SRR7841399_13.6
SRR7866629_13.6
SRR7163869_13.6
SRR9298670_13.6
SRR7167591_13.5
SRR7873592_13.5
SRR9226492_12.1
SRR6807418_12.1
SRR7850130_12.1
SRR7866357_9.3
SRR7873684_9.3
SRR7827106_8.4
BL89-020_1.5
BL89-019_1.5
07-032_1.5
BL87-028_1.2
class 1 internalin InlL
lmo2027 family class 3 internalin

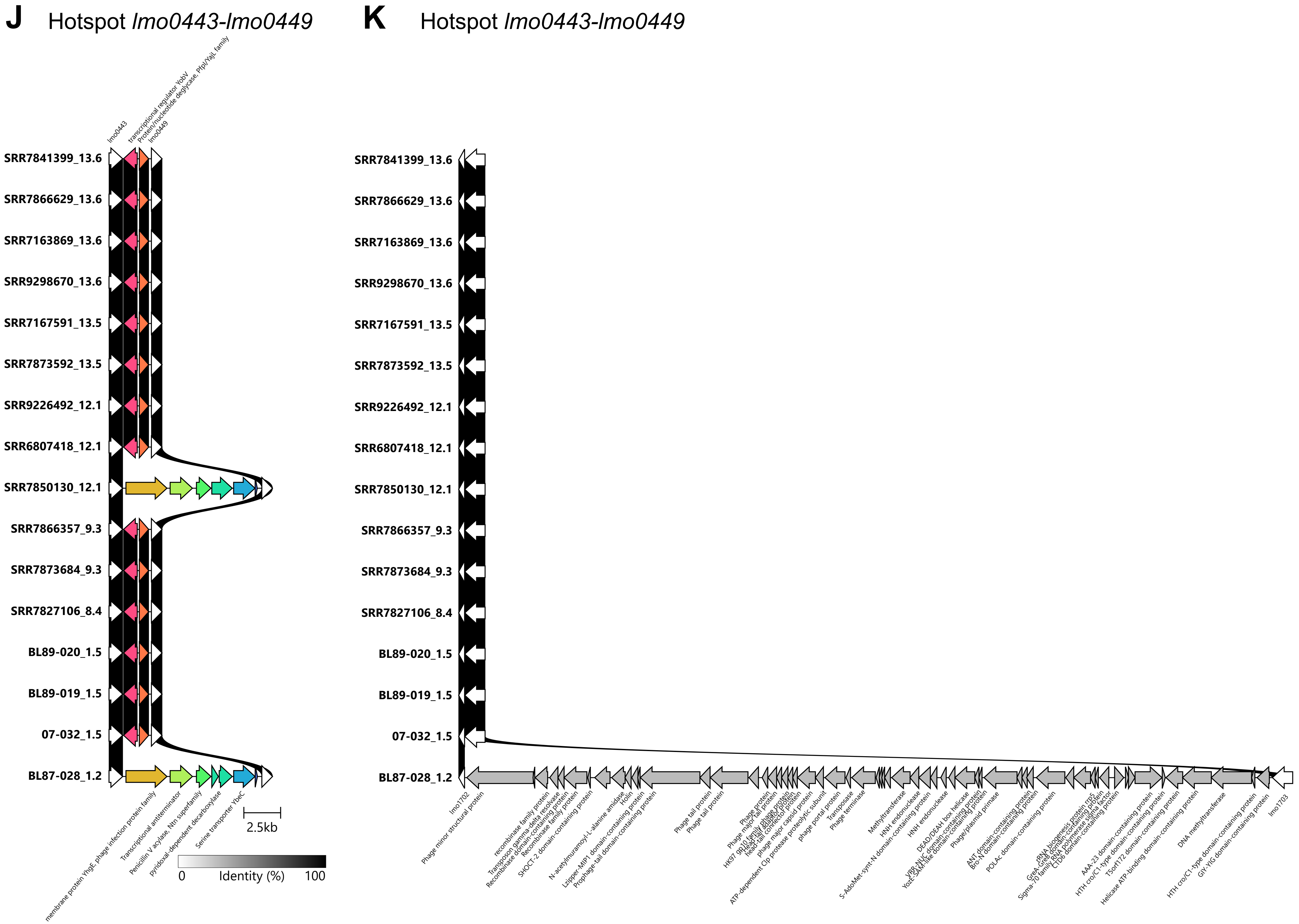

J Hotspot lmo0443-lmo0449
K Hotspot lmo0443-lmo0449
lmo0443
transcriptional regulator YobV
Protein/nucleotide deglycase, PfpI/YajL family
lmo0449
SRR7841399_13.6
SRR7866629_13.6
SRR7163869_13.6
SRR9298670_13.6
SRR7167591_13.5
SRR7873592_13.5
SRR9226492_12.1
SRR6807418_12.1
SRR7850130_12.1
SRR7866357_9.3
SRR7873684_9.3
SRR7827106_8.4
BL89-020_1.5
BL89-019_1.5
07-032_1.5
BL87-028_1.2
membrane protein YhgE, phage infection protein family
Transcriptional antiterminator
Penicillin V acylase, Ntn superfamily
pyridoxal-dependent decarboxylase
Serine transporter YbeC
2.5kb
0
Identity (%)
100
lmo1702
Phage minor structural protein
lmo1703

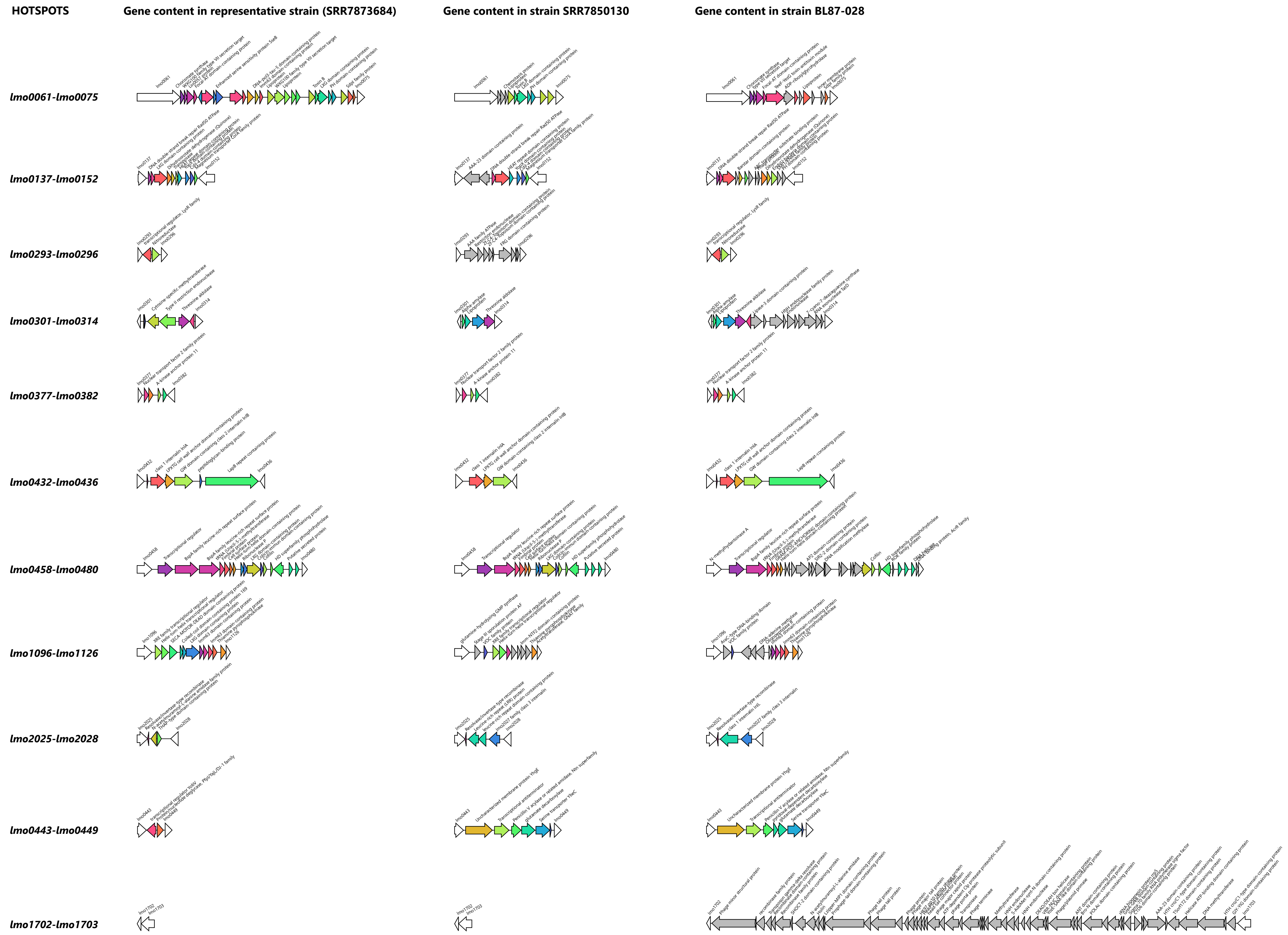
HOTSPOTS
Gene content in representative strain (SRR7873684)
Gene content in strain SRR7850130
Gene content in strain BL87-028
lmo0061-lmo0075
lmo0137-lmo0152
lmo0293-lmo0296
lmo0301-lmo0314
lmo0377-lmo0382
lmo0432-lmo0436
lmo0458-lmo0480
lmo1096-lmo1126
lmo2025-lmo2028
lmo0443-lmo0449
lmo1702-lmo1703

M Prophage insertion site *lmo1750-lmo1751*

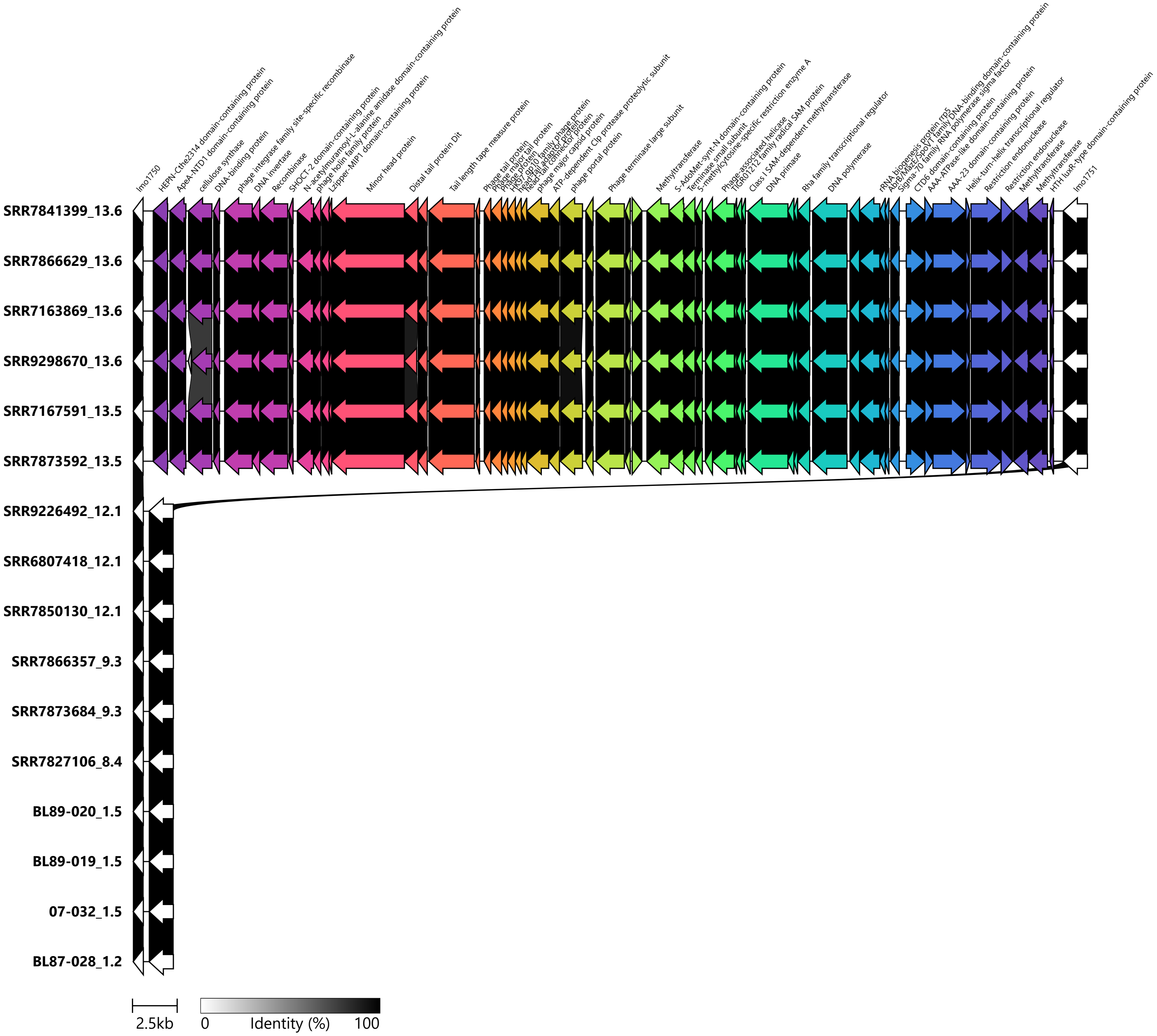

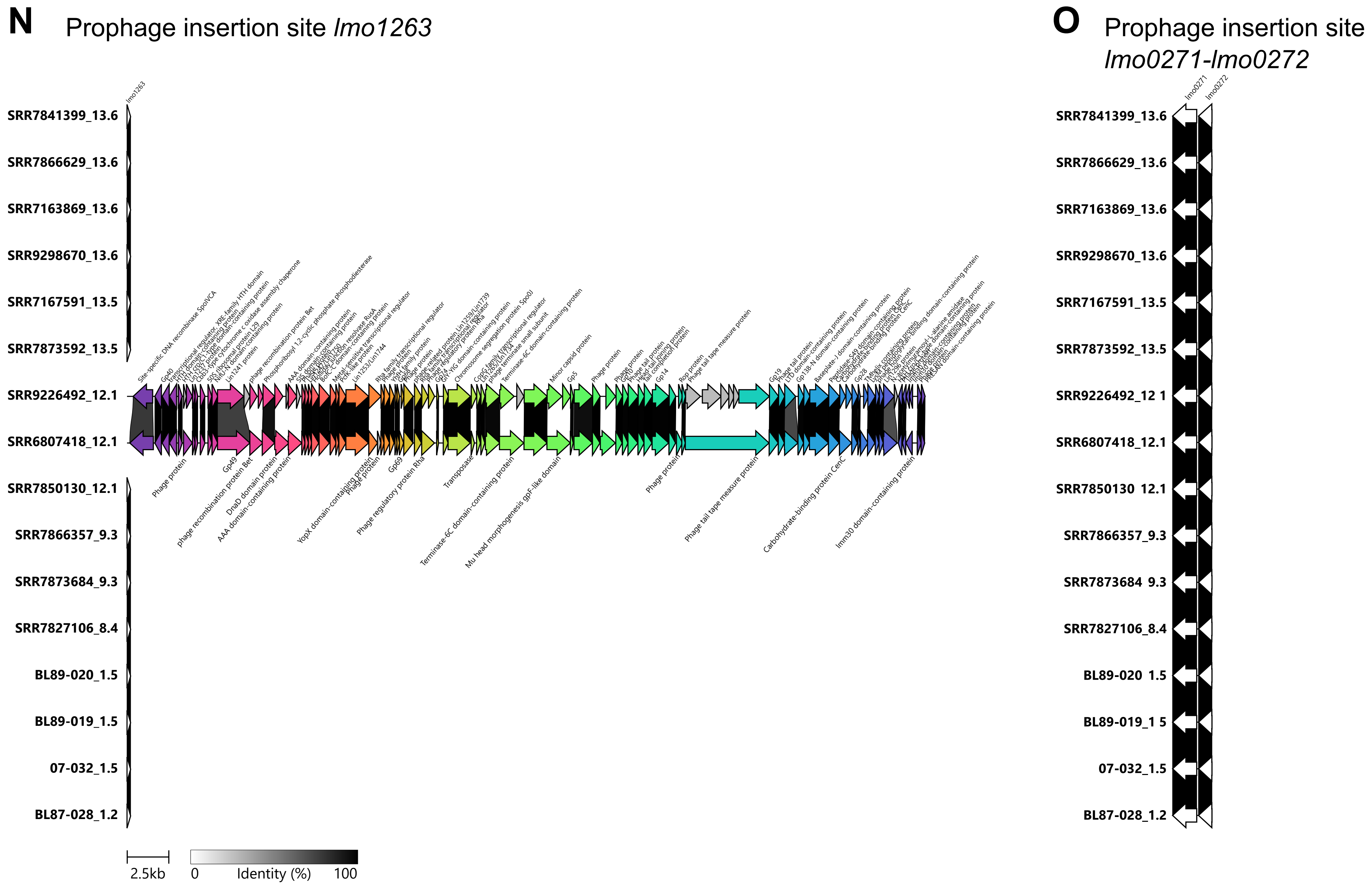
N
Prophage insertion site lmo1263
O
Prophage insertion site lmo0271-lmo0272
lmo1263
lmo0271
lmo0272
SRR7841399_13.6
SRR7866629_13.6
SRR7163869_13.6
SRR9298670_13.6
SRR7167591_13.5
SRR7873592_13.5
SRR9226492_12.1
SRR6807418_12.1
SRR7850130_12.1
SRR7866357_9.3
SRR7873684_9.3
SRR7827106_8.4
BL89-020_1.5
BL89-019_1.5
07-032_1.5
BL87-028_1.2
2.5kb
0
Identity (%)
100

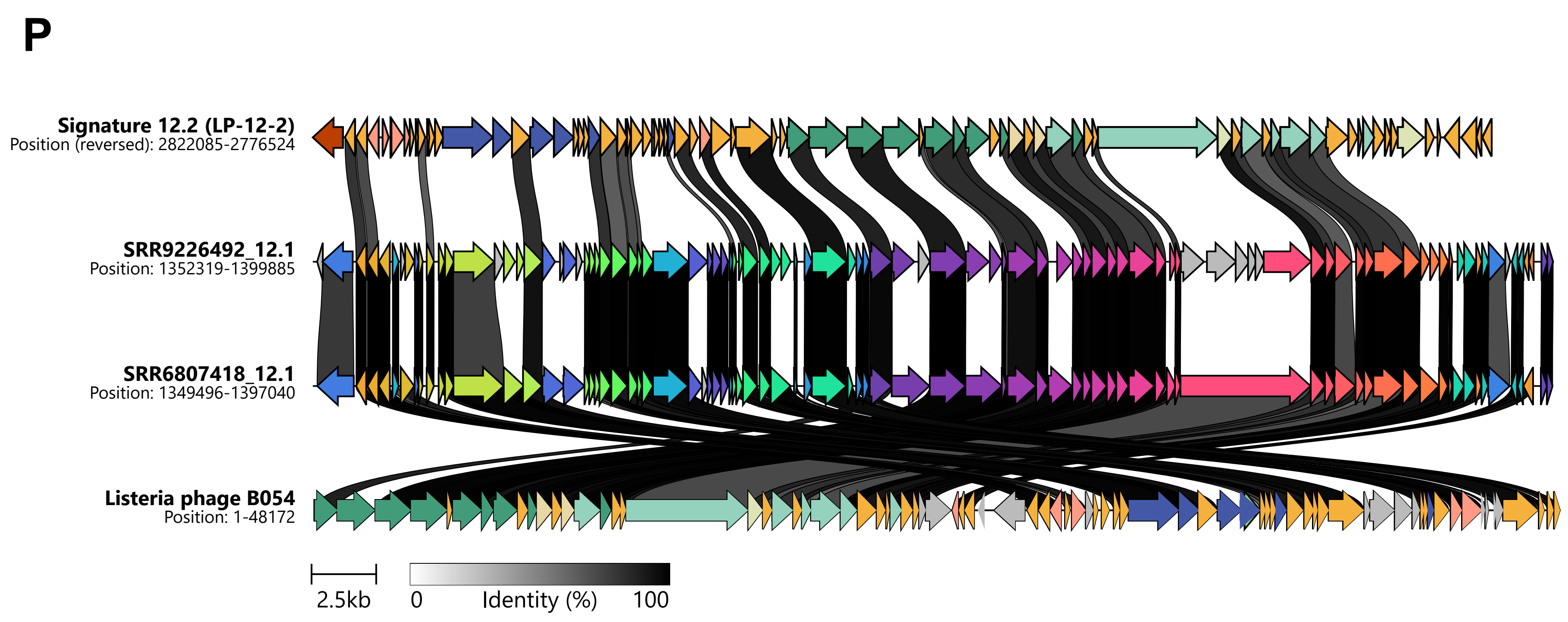

P
Signature 12.2 (LP-12-2)
Position (reversed): 2822085-2776524
SRR9226492_12.1
Position: 1352319-1399885
SRR6807418_12.1
Position: 1349496-1397040
Listeria phage B054
Position: 1-48172
2.5kb
0
Identity (%)
100

98. Müller A, Rychli K, Muhterem-Uyar M, Zaiser A, Stessl B, *et al*. Tn6188 - a novel transposon in *Listeria monocytogenes* responsible for tolerance to Benzalkonium Chloride. PLoS One 2013;8:e76835. 10.1371/journal.pone.0076835
